# SpatialcoGCN: deconvolution and spatial information–aware simulation of spatial transcriptomics data via deep graph co-embedding

**DOI:** 10.1093/bib/bbae130

**Published:** 2024-03-31

**Authors:** Wang Yin, You Wan, Yuan Zhou

**Affiliations:** Department of Biomedical Informatics, School of Basic Medical Sciences, Peking University, 38 Xueyuan Road, Beijing 100191, China; State Key Laboratory of Vascular Homeostasis and Remodeling, Peking University, 38 Xueyuan Road, Beijing 100191, China; Department of Neurobiology, School of Basic Medical Sciences, Neuroscience Research Institute, Peking University, 38 Xueyuan Road, Beijing 100191, China; Department of Neurobiology, School of Basic Medical Sciences, Neuroscience Research Institute, Peking University, 38 Xueyuan Road, Beijing 100191, China; Department of Biomedical Informatics, School of Basic Medical Sciences, Peking University, 38 Xueyuan Road, Beijing 100191, China; State Key Laboratory of Vascular Homeostasis and Remodeling, Peking University, 38 Xueyuan Road, Beijing 100191, China

**Keywords:** spatial transcriptomics, cell type deconvolution, graph-based deep learning, spatial transcriptomics data simulation

## Abstract

Spatial transcriptomics (ST) data have emerged as a pivotal approach to comprehending the function and interplay of cells within intricate tissues. Nonetheless, analyses of ST data are restricted by the low spatial resolution and limited number of ribonucleic acid transcripts that can be detected with several popular ST techniques. In this study, we propose that both of the above issues can be significantly improved by introducing a deep graph co-embedding framework. First, we establish a self-supervised, co-graph convolution network–based deep learning model termed SpatialcoGCN, which leverages single-cell data to deconvolve the cell mixtures in spatial data. Evaluations of SpatialcoGCN on a series of simulated ST data and real ST datasets from human ductal carcinoma *in situ*, developing human heart and mouse brain suggest that SpatialcoGCN could outperform other state-of-the-art cell type deconvolution methods in estimating per-spot cell composition. Moreover, with competitive accuracy, SpatialcoGCN could also recover the spatial distribution of transcripts that are not detected by raw ST data. With a similar co-embedding framework, we further established a spatial information–aware ST data simulation method, SpatialcoGCN-Sim. SpatialcoGCN-Sim could generate simulated ST data with high similarity to real datasets. Together, our approaches provide efficient tools for studying the spatial organization of heterogeneous cells within complex tissues.

## INTRODUCTION

Detailed depiction of gene expression patterns among heterogeneous cell populations from an intricate tissue is critical for the comprehensive understanding of the molecular mechanisms in various physiological and complex disease processes [1]. To this end, single-cell ribonucleic acid (RNA) sequencing (scRNA-seq) and spatial transcriptomics (ST) profiling have become promising complementary experimental approaches to obtaining the single-cell resolution gene expression pattern in tissues [2]. More specifically, scRNA-seq can separate and profile thousands of single cells from one tissue sample simultaneously, but the original spatial organization of these cells is lost by this procedure. Conversely, ST has become a powerful approach to depict gene expression patterns within a tissue’s spatial context. However, several popular ST techniques based on next-generation sequencing are still restricted by their lower spatial resolution in which each spatial sampling spot may cover multiple cells rather than explicit single cells [3–5], while other ST techniques based on *in situ* hybridization and fluorescence microscopy are relatively weaker in the total number of RNA transcripts that they can detect [6–8].

To address the low spatial resolution issue of ST data, various computational ST deconvolution methods have been developed to decompose spatial mixtures of each ST sampling spot into the proportion of each cell type on this spot, by integrating ST data with scRNA-seq annotations, such as CARD [9], Cell2location [10], SpatialDWLS [11], RCTD [12], DestVI [13], Stereoscope [14], Tangram [15], Seurat [16] and SPOTlight [17]. These methods can be roughly categorized into three groups. The first group is regression-based methods. For example, SpatialDWLS and SPOTlight endeavor to estimate cell-type proportions at each spatial location by utilizing regression models. SpatialDWLS employs an enrichment analysis to recognize the cell type at each location, followed by a regression model to estimate the proportion of the chosen cell type [11]. SPOTlight is a deconvolution algorithm that employs the non-negative matrix factorization (NMF) regression algorithm as well as the non-negative least squares (NNLS), where NMF is carried out to identify cell type–specific topic profiles in scRNA-seq references and NNLS is carried out to identify the linking between spot expression profiles in ST data and the topic profiles [17]. CARD is also built upon an NMF model but devises an *ad hoc* conditional auto-regression term to consider spatial correlation of cell type compositions when deconvoluting ST data [9]. Seurat uses canonical correlation analysis (CCA) to embed spatial and scRNA-seq data into a common latent space and projects cells from scRNA-seq data onto the spots of the ST data [16]. The second group is probabilistic model–based methods. For example, Cell2location and RCTD construct on the basis of probabilistic models combined with negative binomial or Poisson distributions. Cell2location is a Bayesian model that can resolve fine-grained cell types in ST data and create comprehensive cellular maps of diverse tissues. It accounts for technical sources of variation and borrows statistical strength across locations, thereby enabling the integration of single-cell and ST with higher sensitivity and resolution than existing tools [10]. RCTD assumes that the observed gene expression in a spot is a linear combination of the reference cell type expression profiles plus technical noises. Using scRNA-seq reference data, RCTD estimates cell-type proportions in each spot by fitting a noisy linear model with differentially expressed genes and platform effects [12]. Stereoscope leverages a model-based probabilistic method to deconvolve the cell mixtures in spatial data, resting on the primary assumption that both spatial and single-cell data follow a negative binomial distribution [14]. Finally, with the rapid progression of deep learning frameworks, several deep learning–based ST deconvolution methods have been established. Tangram employs deep neural network–based non-convex optimization to acquire spatial alignment for scRNA-seq data [15]. DestVI introduces variational inference and latent variable models to explicate the cell type proportions [13].

In order to perform an impartial and comprehensive assessment of various ST deconvolution methods, some benchmarking studies have tested deconvolution methods from multiple aspects [18–21]. Li *et al.*’s benchmarking [18], which largely relied on various simulated ST datasets, has shown that Cell2location and SpatialDWLS outperformed other methods, followed by the probabilistic models RCTD, while the performances of deep learning methods like Tangram and DestVI were shown to be inferior to other types of methods mentioned above. Chen *et al.*’s benchmarking [19] assesses deconvolution performance with either internal reference (i.e. using the single-cell resolution ST dataset itself as the scRNA-seq reference) or external reference (other scRNA-seq datasets from the same tissue) and concludes that RCTD and stereoscope achieve more robust and accurate inferences. Yan *et al*.’s [20] benchmarking is performed on synthetic ST datasets and the real ST dataset of human developing heart and finds cell2loction, RCTD and SpatialDWLS are more accurate than other ST deconvolution methods, based on the evaluation of three metrics: root-mean-square error (RMSE), Pearson correlation coefficient (PCC) and Jensen–Shannon divergence (JSD). Li *et al.*’s benchmarking [21] is one of the most recent one that covers 18 existing cellular deconvolution methods (including three reference-free methods) used in ST. The methods are evaluated on 50 real and simulated datasets, and the results suggest that CARD, Cell2location, Tangram and RCTD are usually the best-performing methods. Although the performance ranking reported in different benchmarking studies varies, these studies have highlighted two common challenges of current deep learning–based ST deconvolution methods. On the one hand, since the evaluated deep learning–based method did not achieve sound rankings in several benchmarking studies, more sophisticated deep learning methods should be developed to more efficiently exploit scRNA-seq and ST data. On the other hand, it is also noteworthy that the simulated ST data used in the benchmarking studies were often generated without specific spatial constraints. Therefore, how to exploit deep learning techniques, which have advantages in dealing with complicated structured data, to enable spatial information-aware simulation is also a critical issue. Spatial information–aware ST data simulation can better mimic the ground truth spatial gene expression pattern and therefore achieves more realistic model training and evaluation.

In this work, we demonstrate that embedding scRNA-seq and ST data into the same space by graph deep learning models could be an efficient way to improve both of the above issues, i.e. ST data deconvolution and simulation. We first developed SpatialcoGCN, a graph deep learning model for ST data deconvolution. With a similar deep graph co-embedding framework, we further proposed a new method SpatialcoGCN-Sim, for the spatial information–aware simulation of ST data.

## METHODS

### Overview of SpatialcoGCN

**Figure 1:**
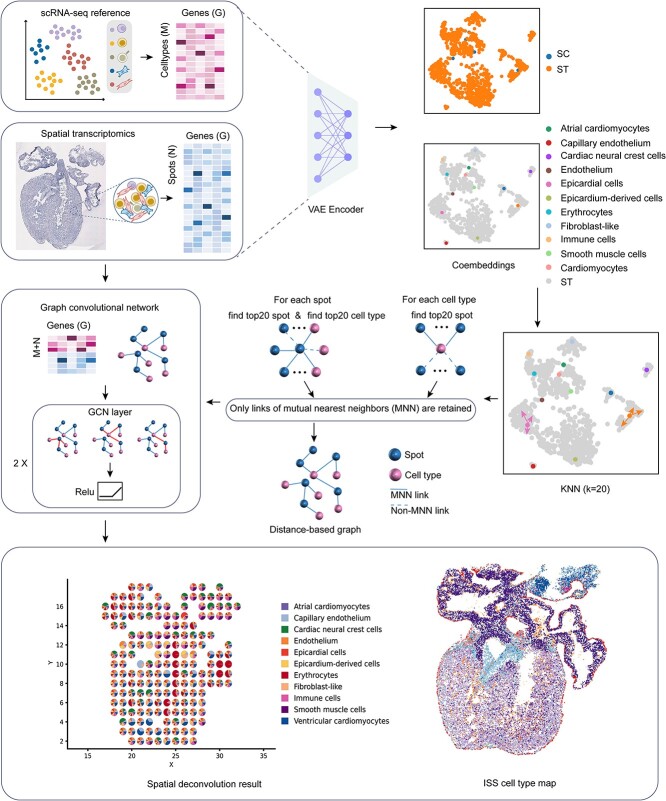
Schematic overview of SpatialcoGCN, an ST data deconvolution method. SpatialcoGCN first transformed scRNA-seq data by summing the expression profiles of cells of the same cell type. The transformed scRNA-seq data and ST data were then projected into a shared embedding space via the encoder architecture of VAE. In this low-dimensional embedding space, SpatialcoGCN established a link graph between cells and spots based on KNN distances. The link graph was merged from spot-to-spot and spot-to-cell subgraphs, and only connections passed the mutual nearest neighbor criterion were retained. Taking the link graph, and the original expression matrix of cell types/spots as the input, a GCN to propagate both transformed scRNA-seq and ST data into the latent layer and solve the mapping matrix that estimate the compositions of different cell types for each spot. Consequently, the compositions of cells in ST data can be accurately learned and predicted.

We first introduce SpatialcoGCN, a deep graph co-embedding-based method to deconvolute ST data into cell type composition and recover the undetected genes per spot. The overview of SpatialcoGCN workflow is shown in Figure 1. The input of SpatialcoGCN is scRNA-seq data along with ST data from the same tissue type or region. However, the input scRNA-seq and ST data do not necessarily profile the identical set of genes. Instead, SpatialcoGCN requires only that these two modalities share part of common genes. By aggregating the gene expression of raw scRNA-seq data according to cell types, the raw scRNA-seq data are transformed as a matrix $C\in{R}^{m\times g}$, where $m$ is the number of cell types and $g$ is the number of genes. Another input, i.e. ST data, is expressed as an ST expression matrix $S\in{R}^{n\times g}$, where $n$ is the number of spots and $g$ is the number of genes. The fundamental hypothesis is that gene expression captured at one spot is contributed by the mixture of cells located at that spot. Therefore, given $C$ and $S$, our objective is to learn a mapping matrix $M\in{R}^{n\times m}$, where the difference between $MC\in{R}^{n\times g}$ and $S$ should be optimized as small as possible.

To achieve this objective, SpatialcoGCN first projects scRNA-seq data and ST data into a common embedding space via the encoder architecture of variational autoencoder (VAE) [22]. Next, SpatialcoGCN establishes a distance-based link graph between the co-embedded data utilizing the *k*-nearest neighbor (KNN) algorithm. In our approach, there is no need to consider connections between cell types. The scRNA-seq expression matrix and ST expression matrix were then stacked based on common genes. Because the link graph in the graph convolution network (GCN) is related to both cell types and spots, the C and S matrices were stacked together so that they can be directly used as input to graph convolution. By taking the concatenated expression matrix $X=\left[{}_S^C\right]\in{R}^{\left(m+n\right)\times g}$, along with the link graph as input, SpatialcoGCN employs a self-supervised GCN to learn the aforementioned mapping matrix and therefore obtains per spot cell type compositions and the spatial distribution of the expressions of undetected transcripts in ST data. During the graph convolution iteration in GCN, the value of each spot is the weighted sum of itself and the values of the connected neighbors (cells and spots) in the link graph. Based on this, the topological relationship between scRNA-seq data and ST data can be fully utilized to generate the mapping matrix.

### Preprocessing of input scRNA-seq and ST datasets

All scRNA-seq and ST datasets were subjected to analysis using the Scanpy package [23]. For scRNA-seq datasets, the datasets were preprocessed through the following procedures: cells with high mitochondrial gene content were eliminated, and the expression matrix was normalized by using sc.pp.normalize_total() from Scanpy package to correct for library size so that the sum of expression levels per cell is 1. For ST datasets, we keep the original count matrix as is, since the expression of one gene in one spot is related not only to the average gene expression level in this spot but also to the number of cells captured by this spot. We excluded cells or spots that expressed less than five genes in the dataset and filtered out genes detected in fewer than one cell or spot. Next, we dropped cell types containing less two cells on scRNA-seq data. Given that 10X Visium data would suffer from prominent noise for less expressed genes, we opted not to map the entire transcriptome, but instead screened out the expression profiles of top 200 genes for each cell type as the training set.

The selection of the number of marker genes and normalization strategy has been investigated through ablation experiments. Firstly, since the selection of marker genes is critical to deconvolution performance, we considered several thresholds for marker genes. Specifically, we pooled the top 50, top 100, top 200 and top 500 marker genes for each cell type. To accommodate different numbers of detectable genes for different cell types, we also tried an alternative scenario by pooling the top 0.5%, top 1% and top 5% marker genes for each cell type based on the total number of detectable genes of each cell type. The results showed that the performance of SpatialcoGCN exhibited consistent enhancement as the marker gene number increased from the top 50 to the top 200, but dropped at the top 500, indicating the top 200 as the optimal choice (Supplementary Figure 1). As for the proportional thresholds, most proportional thresholds do not show better performance than the top 200 threshold, except that models using the top 5% marker genes show slightly better performance than models using the top 200 only for a few metrics, while the cost is obviously increased computation time (Supplementary Figure 1). Taken together, we decided to choose the top 200 genes as the recommended criterion for choosing marker genes. Secondly, before the scRNA-seq expression matrix and ST expression matrix were concatenated, we also need to consider whether it is necessary to normalize the two data so that they are on the same scale. Four configurations were tested (i.e. none normalized, scRNA-seq normalized, ST normalized and both normalized). According to the results, the scRNA-seq normalized configuration could get the best results (Supplementary Figure 2). Therefore, we only normalized the scRNA-seq expression matrix but kept the ST matrix as is.

### VAE co-embedding algorithm

VAE [22] is a deep learning method that can learn low-dimensional latent representations from high-dimensional data. VAE is beneficial for extracting the essential characteristics of cells and reducing technical noise and batch differences that are often used to eliminate batch effects and data integration of scRNA-seq data. VAE has been adopted by several scRNA-seq data processing methods. For example, Scvi-tools [24] applies a conditional VAE framework to model the inherent distribution of the input single-cell data for data integration. SCALEX applies a VAE to project the different batches of single-cell datasets into the same batch-invariant low-dimensional embeddings by learning a batch-free encoder and a batch-specific decoder simultaneously [25]. Besides, VAE can flexibly adjust the dimension and complexity of the latent space to adapt to data of different scales and heterogeneity, thus ensuring the scalability and accuracy of the integration between scRNA-seq and ST-seq data.

SpatialcoGCN applied VAE [22] to project scRNA-seq data and ST data into the same low-dimensional embedding space by learning encoder and decoder simultaneously. In the encoder, VAE took the combined expression matrix of scRNA-seq data and ST data $x\in{R}^{\left(m+n\right)\times g}$ as a whole mixture distribution without distinguishing their data sources. Based on this combined expression matrix, VAE extracted the mean $\mu$ and variance ${\sigma}^2$ of the latent representations $z$ in a 30-dimension embedding space to learn the global data structure. Similar to SCALEX [25], a standard Gaussian distribution is used for $z$, while the approximate distribution of $z$ is re-parameterized by $z=\mu +{\sigma}^2\ast I$, where $I$ is sampled from a standard normal distribution. In the decoder, VAE maps the latent representation back to its original profile.

During training, the parameters of encoder $q\left(z|x\right)$ and decoder $p\left(x|z\right)$ was optimized by maximizing a lower bound on the log-likelihood of expression profile $x$, called the evidence lower bound (ELBO):


(1) \begin{align*} {\text{log}} \ p(x)&={\text{log}}\int p\left(x,z\right) dz \ \nonumber \\ &\ge E_{q\left(z|x\right)}\left[{\text{log}}\ \frac{p\left(x,z\right)}{q\left(z|x\right)}\right]\ \nonumber\\ &=E_{q\left(z|x\right)}\left[{\text{log}} \ p\left(x|z\right)\right]-{D}_{KL}\left(q\left(z|x\right)\parallel p(z)\right)\ \nonumber \\ &={L}_{ELBO}(x)\ \end{align*}

where ${D}_{KL}$ denotes the Kullback–Leibler (KL) divergence and ${L}_{ELBO}(x)$ denotes the likelihood of ELBO.

After training, the encoder was generalized to scRNA-seq data and ST data for globally mapping different sources of datasets into the same embedding space. It is also noteworthy that only the latent representation from the encoder part of VAE is required by the subsequent steps. VAE embeddings are only used to construct the link graph, and the input matrix to GCN is still the original expression matrix (Figure 1).

### Generating link graph in co-embedding space

In the low-dimensional co-embedding space, we identified the mutual nearest neighbors among nodes representing cells and spots. Specifically, the KNN algorithm was used to find the *k*-nearest data nodes to a given query nodes (default *k* is 20). We required that two nodes must be mutual nearest neighbors, and the nearest neighbors between two cell types were not considered. In other words, we only considered connections between cell types and spots as well as connections between spots. To achieve this, we first calculated the sub-link graph between cells and spots and the sub-link graph between spots respectively and then combined the two sub-link graphs into one (Figure 1). Specifically, for each spot, we first find its top 20 neighbor spots and top 20 neighbor cell types. If the number of cell types is less than 20, the spot will connect to all cell types. For each cell type, we also find its top 20 neighbor spots. Then, we calculate the sub-link graph between cells and spots and the sub-link graph between spots, respectively. Only cell types or spots that are in the top 20 nearest neighbors of each other will be connected, to ensure the mutual nearest neighbor criterion. Finally, we merge these two sub-link graphs into the final link graph.

By procedure, the final link graph was represented by the weighted adjacent matrix $A\in{R}^{a\times a}$, where $a=m+n$ denotes the total number of cells and spots. We used the Euclidean distance calculated by the KNN algorithm between nearest neighbor nodes, while only keeping the distance between nodes with connected edges in the final link graph. The distance was taken as the connection weight after normalization by


(2) \begin{equation*} {\begin{array}{c}{w}_{ij}=1-\displaystyle \frac{d_{ij}}{\max (d)}\ \end{array}} \end{equation*}

where ${w}_{ij}$ is the connection weight between node $i$ and node $j$ in the link graph, ${d}_{ij}$ is the Euclidean distance between node $i$ and node $j$ and $\max (d)$ is the maximum Euclidean distance among nodes. It is noteworthy that the mutual nearest neighbors of cells and spots were selected before converting the distances into weights; therefore, the calculation in Equation (2) is insensitive to outliers (since the outliers can hardly pass the mutual nearest neighbor filter).

### Co-graph convolution network

In recent years, the GCN has been demonstrated to be promising in utilizing intrinsic topological information of data to improve model performance. The topological relations inside the data, such as cell-to-spot and spot-to-spot connections, can be effectively utilized by GCN, which provides crucial information about the relations between the observed gene expression patterns and associated cell types at spots. DSTG [26] is a similarity-based semi-supervised GCN model that can recover cell-type proportions in each ST spot based on the linking between spots and pseudo-spots that are aggregated from cells in scRNA-seq reference. However, DSTG does not perform very well in benchmarking studies [18–21], indicating that more cohesively embedding and linking of scRNA-seq and ST data are required.

In this study, we embedded scRNA-seq and ST data into the same space and exploited GCN to cooperatively model the cell type-to-spot and spot-to-spot links. In other words, to predict the per-spot cell type proportions, we utilized the GCN on the graph *G* = (*V*, *E*), where *V* is the set of nodes representing cells and spots and *E* is the edge set representing the connection in the aforementioned link graph. Explicitly, the co-GCN model required two inputs. One input was the stacked expression matrix $X=\left[{}_S^C\right]\in{R}^{\left(m+n\right)\times g}$ that integrated transformed scRNA-seq data $C\in{R}^{m\times g}$ and ST data $S\in{R}^{n\times g}$, where $m$ is the number of cell types, $n$ is the number of spots and $g$ is the number of common genes shared between the scRNA-seq cell type markers and the detectable genes in ST data. Another input was the weighted adjacent matrix $A$ mentioned above.

Based on the stacked expression matrix $X$ and the link graph structure $A$, the co-GCN model was constructed with two convolutional layers. The number of rows of *X* was equal to the number of cell types plus the number of spots, and the columns were the feature dimensions. During the convolution process, only the feature dimensions were iteratively updated, but the number of rows of the matrix was not changed. The layer-wise update rule [27] of GCNs can be applied to features as


(3) \begin{equation*} {\displaystyle \begin{array}{c}{X}^{\left(l+1\right)}=\sigma \left({\tilde{D}}^{-\frac{1}{2}}\tilde{A}{\tilde{D}}^{-\frac{1}{2}}{X}^{(l)}{W}^{(l)}\right)\ \end{array}} \end{equation*}

where $\tilde{A}=A+I$ is the link graph with additional self-loops *I* to keep identity features, $\tilde{D}$ is the diagonal degree matrix of $A$, ${W}^{(l)}$ denotes a learnable weight matrix at layer $l$ of a network and $\sigma \left(\cdot \right)$ is an activation function. The term ${\tilde{D}}^{-\frac{1}{2}}\tilde{A}{\tilde{D}}^{-\frac{1}{2}}{X}^{(l)}$ can be intuitively interpreted as an approximate spatial mean feature aggregation from the direct neighborhood followed by an activated linear layer.

The output of final layer $Y=\left[{}_{Y_{st}}^{Y_{sc}}\right]\in{R}^{\left(m+n\right)\times m}$ contains the celltype-to-celltype mapping matrix *Y_sc_* and the spot-to-celltype mapping matrix *Y_st_*. Since only the *Y_st_* part is related to the ST deconvolution result, this ST-related part was extracted as the predicted mapping matrix $M\in{R}^{n\times m}$, such that ${M}_{ij}\ge 0$ is the proportions of cell type$j$ in spot $i$. In this matrix, each spot needs to have a proportional constraint:


(4) \begin{equation*} \sum_j^m{M}_{ij}=1\ \end{equation*}

To achieve the above purpose, we defined the normalization function:


(5) \begin{equation*} {M}_{ij}=\frac{{\tilde{M}}_{ij}}{\sum_l^m{\tilde{M}}_{il}} \end{equation*}

After normalization, we ensure that $0\le{M}_{ij}\le 1$.

To learn the mapping matrix, the following loss function should be minimized:


(6) \begin{equation*}\kern-.6pc {\displaystyle \begin{array}{c}L=\frac{1}{g}\left(\sum_i^g\left(1- PCC\left({(MC)}_{\ast, i},{S}_{\ast, i}\right)+1- COSSIM\left({(MC)}_{\ast, i},{S}_{\ast, i}\right)\right)\right) \\{}+\frac{1}{n}\left(\sum_j^n\left(1- PCC\left({(MC)}_{j,\ast },{S}_{j,\ast}\right)+1- COSSIM\left({(MC)}_{j,\ast },{S}_{j,\ast}\right)\right)\right)\end{array}} \end{equation*}

where $PCC$ is the Pearson correlation coefficient, $COSSIM$ is the cosine similarity function and $\ast$ indicates the index. The first term ensures that the predicted expression for each gene over the spots is proportional to the expected gene expression over the spots. The second term ensures that the predicted gene expression needs to be proportional to the expected gene expression for each spot. Note that the PCC and COSSIM were both used to measure the similarity between two vectors in the loss function, where the PCC is often more sensitive to signals while COSSIM is often more robust to noise. Through the ablation experiment, we found that these two similarities could complement each other. More specifically, three configurations were tested (i.e. using the PCC alone, using COSSIM alone and using both the PCC and COSSIMs in the loss function) on 12 spatial information–aware simulated ST datasets and 20 regular simulated ST datasets. Finally, we found that the PCC plus COSSIM indeed performed the best (Supplementary Figure 3).

### Generation of spatial information–aware simulated ST datasets through SpatialcoGCN-Sim

SpatialcoGCN-Sim is a general and flexible computational framework for simulating gene expression count data for ST. SpatialcoGCN-Sim primarily performs reference-based simulations that need both scRNA-seq data and reference ST data as its inputs. The input data are described in Supplementary Table 1. SpatialcoGCN-Sim relies on the reference ST data to obtain the spatial coordinates of the measured locations. Specifically, we used VAE to map the gene expression in each cell and spot into the low-dimensional embedding space to derive the latent representation. KNN was employed to identify the two nearest spots in reference ST data for each cell in the low-dimensional space; then, the predicted two-dimensional (2D) coordinates of the cell were set as the midpoint coordinates of the two spots. As a result, each cell is assigned to 2D coordinates. Finally, we used the hexagonal grid to blur the spatial expression pattern, mimicking the low-spatial-resolution spots in ST data. The radius of the spots, in other words, the spatial resolution of the simulated datasets, can be customized by adjusting the size of the hexagonal grid. To ensure each gridded ‘spot’ contains 4–15 cells (similar to the ST datasets generated by the 10X Visium or ST approaches), we randomly transferred extra cells (if any) from each spot to its six neighbor spots.

In the above process, we set *k* = 2 when running KNN, in order to identify the two nearest spots in reference ST data for each cell in the low-dimension space and predict the coordinates of each cell based on these two nearest spots. To better illustrate the involvement of spatial information during this process, we conducted a comparative analysis of the performance of *k* = 1 and *k* = 2 (Supplementary Figure 4). For dataset 8, when setting *k* = 1, the predicted locations of the cells in scRNA-seq data were exactly coincident with the coordinates of the spots in the reference ST data, which reduced the diversity of new spots (Supplementary Figure 4A–C). Conversely, setting *k* = 2 led to the generation of a substantial number of new spots (Supplementary Figure 4D–F). At the same time, the spatial organization pattern of cells also becomes more evident (i.e. the distribution of the same cell type becomes more concentrated). Similar tendency can also be observed in dataset 12 (Supplementary Figure 4G–L). Notably, based on the spatial continuity of spots, *k* = 2 would be a better choice when emphasizing the spatial topology of the spots (Supplementary Figure 4F and L).

To further quantitatively evaluate the advantage of our spatial information–aware simulation method, we defined a metric called spatial expression correlation. This metric quantified the similarity of gene expression profiles between each spot and its spatial neighbors in the simulated ST datasets. We first identified the spatial neighbors of each spot within a given radius range and then calculated the PCC between the expression profile of each spot and that of its neighbor spots. We used the coverage range per spot in the ST data as the base radius *r* and consider different folds of the base radius (i.e. *r*, *2r* and *4r*) as the radius range to define neighbor spots. Finally, the spatial expression correlation was the average of the PCC values of all spots.

To avoid confusion, we hereafter refer to the simulated ST datasets by SpatialcoGCN-Sim as spatial information–aware simulation datasets and the simulated ST datasets by RCTD and Stereoscope as regular simulated ST datasets.

### Generation of regular simulated ST datasets via simulation algorithm introduced by RCTD and Stereoscope

The 20 regular simulated datasets (listed in Supplementary Table 2) were generated according to the algorithm introduced by RCTD [12] and Stereoscope [14]. This method directly simulates the ST dataset from scRNA-seq data. Briefly, for each spot in the simulated dataset, we first sampled cell numbers in a uniform distribution in the range of 5–15 and sampled the number of cell types in a uniform distribution in the range of 2–6. Then, we randomly assigned cells from each cell type to the spot. When performing the deconvolution experiments, for each experiment, we used one scRNA-seq to generate the simulated ST data and used another scRNA-seq data that were from the same tissue type or region and had the same cell type annotation as the reference scRNA-seq, in order to keep the simulated ST data independent to the reference scRNA-seq. The intersect genes between the simulated ST data and the reference scRNA-seq data were retained for the deconvolution experiments. Finally, the gene expression values of each spot were obtained by summing the gene expression values of all cells mapped to this spot. The proportion of a cell type at each spot could be calculated by counting the number of cells corresponding to the cell type. Note that some deconvolution methods such as CARD require the coordinate location of the ST spots as input. Therefore, we sequentially assigned a positive integer coordinate to each spot of the simulated data, such as (1,1), (1,2), (2,1) and (2,2). That is to say, the coordinates of the first simulated spot are (1,1), the coordinates of the second simulated spot are (1,2) and so on. To prevent all spots from being distributed in one row, we limited the maximum column coordinate as 40.

### Evaluation on noisy simulation datasets

Our assumption is that the gene expression in one spot is only contributed to by the mixture of cells located at this spot. But in real-world conditions, the RNAs may spread to neighboring spots during the permeation step in ST experiments. Our spatial information–aware simulation enables us to model such neighbor-derived noises. Explicitly, to simulate this phenomenon, we added noise to the spatial information–aware simulation ST data based on the following formula:


(7) \begin{equation*} { \begin{array}{c}{\overline{E}}_i=\left(1-\mathrm{\alpha} \right)\ast{E}_i+\alpha \ast \displaystyle\frac{\sum_j^J{E}_j}{J} \end{array}} \end{equation*}

where ${E}_i$ and ${\overline{E}}_i$ are, respectively, the original and new expression values of spot $i$, ${E}_j$ is the expression value of spot $j$, which is the neighbor of spot $i$, and $J$ is the number of neighbor spots of spot $i$. $\alpha$ represents the degree of influence by neighbors.

We simulated two scenarios of noise addition, where 25% or 50% of the spots were affected by neighbor spots. For each scenario, we varied $\alpha$ to 10% or 20%, resulting in four sets of noisy ST datasets with different configurations for each input simulation dataset.

### Performance evaluation metrics

Similar to Li *et al.* [18], we used the following six metrics to evaluate the performance of the deconvolution methods for simulated data.

1. PCC. The PCC value was calculated using the following equation:


(8) \begin{equation*} { \begin{array}{c} PCC=\displaystyle\frac{E\left[\left({\tilde{y}}_i-{\tilde{\mu}}_i\right)\left({y}_i-{\mu}_i\right)\right]}{{\tilde{\sigma}}_i{\sigma}_i}\end{array}} \end{equation*}

where ${\tilde{y}}_i$ and ${y}_i$ are, respectively, the proportion of various types of cells of spot $i$ in the predicted result and the ground truth, ${\mu}_i$ and ${\tilde{\mu}}_i$ are the average proportion of various types of spot $i$ in the ground truth and the predicted result and ${\sigma}_i$ and ${\tilde{\sigma}}_i$ are the standard deviation of the proportion of spot $i$ in the ground truth and the predicted result. For one spot, a higher PCC value indicates better prediction accuracy.

2. Structural similarity index (SSIM). The SSIM value was calculated using the following equation:


(9) \begin{equation*} { \begin{array}{c} SSIM=\displaystyle\frac{\left(2{\tilde{\mu}}_i{\mu}_i+{\alpha}^2\right)\left(2\mathit{\operatorname{cov}}\left({y}_i,{\tilde{y}}_i\right)+{\beta}^2\right)}{\left({\tilde{\mu}}_i^2+{\mu}_i^2+{\alpha}^2\right)\left({\tilde{\sigma}}_i^2+{\sigma}_i^2+{\beta}^2\right)}\end{array}} \end{equation*}

where the definitions of ${\mu}_i$, ${\tilde{\mu}}_i$, ${\sigma}_i$ and ${\tilde{\sigma}}_i$ are same to those for calculating the PCC; ${\tilde{y}}_i$ and ${y}_i$ are scaled between 0 and 1; $\alpha$ and $\beta$ are 0.01 and 0.03, respectively; and $\mathit{\operatorname{cov}}\left({y}_i,{\tilde{y}}_i\right)$ is the covariance. For one spot, a higher SSIM value indicates better prediction accuracy.

3. COSSIM. The COSSIM value was calculated using the following equation:


(10) \begin{equation*} { \begin{array}{c} COSSIM=\displaystyle\frac{\sum_{j=1}^m{\tilde{y}}_{ij}{y}_{ij}}{\left\Vert{\tilde{y}}_i\right\Vert \times \left\Vert{y}_i\right\Vert}\end{array}} \end{equation*}

where $\left\Vert \bullet \right\Vert$ denotes the ${L}_2$ norm, $m$ is the number of cell types, ${\tilde{y}}_{ij}$ is the predicted cell-type proportion for the cell type $j$ in spot $i$, ${y}_{ij}$ is the ground-truth cell-type proportion for the cell type $j$ in spot $i$ and the definitions of ${\tilde{y}}_i$ and ${y}_i$ are same to those for calculating the PCC. For one spot, a higher COSSIM value indicates better prediction accuracy.

4. RMSE. The RMSE was computed for each spot between the deconvolved and matched ground-truth cell-type proportions in the ST dataset:


(11) \begin{equation*} { \begin{array}{c} RMSE=\sqrt{\displaystyle\frac{\sum_{j=1}^m{\left({\tilde{y}}_{ij}-{y}_{ij}\right)}^2}{m}}\end{array}} \end{equation*}

where the definitions of parameter are same to those for calculating the COSSIM. For one spot, a lower RMSE value indicates better prediction accuracy.

5. JSD. The JSD value was calculated using the following equation:


(12) \begin{equation*} { \begin{array}{c} JSD=\frac{1}{2} KL\left({\tilde{y}}_i|\displaystyle\frac{{\tilde{y}}_i+{y}_i}{2}\right)+\displaystyle\frac{1}{2} KL\left({y}_i|\frac{{\tilde{y}}_i+{y}_i}{2}\right)\end{array}} \end{equation*}

(13) \begin{equation*} { \begin{array}{c} KL\left({p}_i\parallel{q}_i\right)=\sum\limits_{j=0}^M\left({p}_{ij}{\text{log}}\ \displaystyle\frac{p_{ij}}{q_{ij}}\right)\end{array}} \end{equation*}

where $KL\left({p}_i\parallel{q}_i\right)$ is the KL divergence between two probability distribution ${p}_i$ and ${q}_i$. For one spot, a lower JSD value indicates better prediction accuracy.

6. Average rank score (ARS). We defined ARS by aggregating PCC, SSIM, COSSIM, RMSE and JSD to evaluate the relative accuracy of the methods for each benchmarking dataset. After calculating above five metrics, we sorted the PCC, COSSIM and SSIM values of the methods in ascending order and sorted the RMSE and JSD values of the integration methods in descending order. The ranking of one metric for one method is represented by R. Finally, we calculated the average value of all five rankings to obtain the ARS value of each method:


(14) \begin{equation*} {\displaystyle \begin{array}{c} ARS=\frac{1}{5\times M}\left({\mathrm{R}}_{PCC}+{\mathrm{R}}_{SSIM}+{\mathrm{R}}_{COSSIM}+{\mathrm{R}}_{RMSE}+{\mathrm{R}}_{JSD}\right)\end{array}} \end{equation*}

where $M$ is the number of compared methods. For a dataset, the method with the highest ARS value had the best performance among the integration methods.

### Preparation of and evaluation on blurred MERFISH and Stereo-seq datasets

MERFISH datasets [8], image-based high-resolution ST data, contain gene expression profiles, spatial locations and cell-type annotations of individual cells. MERFISH datasets could be used to simulate low-resolution spots by binning the cells with a unified square size, and the ground truth is naturally the cell type proportion in each bin. To evaluate the robustness of SpatialcoGCN, three resolutions were simulated with MERFISH datasets by using different binning sizes of 20, 50 and 100 μm. That is to say, we blurred cell-resolution spots into low-resolution spots by binning them using 20 μm × 20 μm square (bin20), 50 μm × 50 μm square (bin50) and 100 μm × 100 μm square (bin100) and summing up the gene expression profiles inside each bin. These blurred MERFISH datasets were used as the input for deconvolution to check whether the algorithms could correctly re-capture the ground-truth cell composition in each bin.

The high-resolution Stereo-seq [28] ST data can also be exploited to generate low resolution data for performance evaluation in a similar fashion. Here, we utilized the Stereo-seq mouse brain dataset from Chen *et al*. [28] (some other Stereo-seq data contain too many spots to be analyzed by several pervious deconvolution methods and thus cannot be used for performance comparison). We blurred cell-resolution spots into low-resolution spots by binning them using 100 × 100 coordinate units (bin100) and summing up the gene expression profiles inside each bin. The blurred Stereo-seq dataset was used as the input for deconvolution to check whether the algorithms could correctly re-capture the cell composition in each bin.

### Acquisition and analysis of human ductal carcinoma *in situ* data

The ductal carcinoma *in situ* (DCIS) scRNA-seq data and ST data were obtained from CellTrek [29]. For the scRNA-seq data, six main cell types have been annotated, including normal epithelial cells, tumor cells, endothelial cells, fibroblast cells, myeloid cells and natural killer (NK)/T cells. According to copy number profiles from the scRNA-seq data, the study identified three main tumor subclones (i.e. subclone1–3) across all tumor cells; therefore, this scRNA-seq can also be annotated with eight cell types (five non-tumor cell types plus three tumor cell subclones). We deconvoluted the ST data with six and eight cell types, respectively, to test whether SpatialcoGCN can robustly work when the cell type number varies. For ST data, to test if the deconvolution model can accurately identify tumor (or tumor subclone) spots, the ST spots covering the tumor areas were selected based on the matched histopathology image.

### Collection and analysis of scRNA-seq and ST data of developing human heart

The data pertaining to the developing human heart were obtained from a previous study [30]. The study provided four, nine and six heart sections from three timepoints, 4.5–5, 6.5 and 9 post-conception weeks (PCW), respectively, which were profiled by ST technology. They also provided a 6.5 PCW cell-type map with single-cell resolution generated by the *in situ* sequencing (ISS) technology. These ISS data are single-cell resolution ST data that were used as the ground truth to verify SpatialcoGCN. The data processing method is the same as the previous study [19]. First, genes expressed in at least three cells are kept in the analysis. Cells that are co-located in the same 454 × 424 square pixel area were pooled as one ST spot. The scRNA-seq profiles of the 6.5 PCW tissue sample were generated through the 10X Genomics Chromium platform. The scRNA-seq data were clustered and annotated using the markers provided by the original study [30]. The cell types in the ISS data and reference scRNA-seq data are identical. Finally, the scatter pie plot was utilized to visualize the results of deconvolution.

### Collection and analysis of scRNA-seq and ST data of mouse brain

The single-cell dataset was downloaded from http://mousebrain.org, where only the cells originating from hippocampus were used because of its less ambiguous cell type annotations [31]. We used the ‘Class’ identifiers for each cell as the cell type labels, and eight cell types were finally identified. The ST data of mouse brain was directly retrieved from Andersson *et al*. [14]. The ST spots covering different brain regions were selected based on the matched histology image.

### Evaluation of the capability to predict the spatial distribution of undetected transcripts

We also compared the performance of SpatialcoGCN and previous methods for predicting the spatial distribution of undetected transcripts via 10-fold cross-validation on five matched datasets (matched datasets mean that scRNA-seq reference data and ST data were from the same tissue type or region). The source of these matched datasets is described in more details in Supplementary Table 3. By performing 10-fold cross-validation, we first divided all genes covered by ST data into 10 fractions, and iteratively used the expressions of nine fractions of the genes as the input to train the mapping matrix between scRNA-seq data and ST data, and used the expressions of the remaining one fraction of genes as the testing samples. Since the spatial localization of the remaining one fraction is unknown to the trained model, the consistency between the predicted spatial distribution of these genes and their real spatial distribution can be used to evaluate the capability to predict the spatial distribution of undetected transcripts. We aggregated the predictions from all 10 fractions and compared them to the real ST data via the performance evaluation metrics described above (i.e. PCC, SSIM, COSSIM, RMSE, JSD and ARS).

## RESULTS

### Performance of SpatialcoGCN on the regular simulated ST datasets

Since previous benchmarking studies were often performed on regular simulated ST data, which have no specific concerns in spatial coordinates, we first simulated 20 ST datasets with various parameter configurations (Supplementary Table 2; see also Methods), based on the algorithm introduced by RCTD [12] and Stereoscope [14]. Among these datasets, each simulated spot contains 5–15 cells sampled from the scRNA-seq data and the number of cell types in each spot ranges in 2–6.

We evaluate the performance of deconvolution methods in predicting the cell type composition of spots on these regular simulated datasets. Nine popular ST deconvolution methods, including CARD [9], Cell2location [10], SpatialDWLS [11], RCTD [12], Stereoscope [14], Tangram [15], DestVI [13], Seurat [16] and SPOTlight [17] were included in the comparison. PCC, SSIM, COSSIM, RMSE and JSD metrics were employed as the performance evaluation metrics for method comparison. For one spot, a higher PCC, SSIM or COSSIM or a lower RMSE or JSD indicates better performance. Similar to Li *et al*. [18], we also introduced ARS by aggregating the above five metrics.

**Figure 2:**
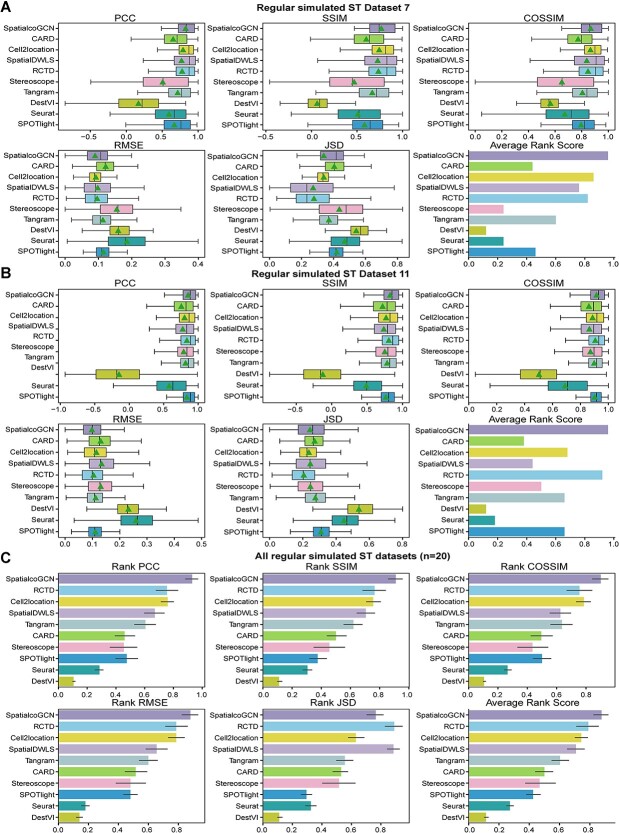
Evaluation of deconvolution performance on regular simulated ST datasets. Comparing the performance of SpatialcoGCN against nine other methods with the capability of deconvoluting cell types for each ST spot, we present our results as boxplots and bar plots. Boxplots: center line, median; box limits, upper and lower quartiles; triangle: mean. Bar plots: data are presented as mean values ± 90% confidence intervals. (**A**) The boxplots of PCC, SSIM, COSSIM, RMSE, and JSD, as well as the bar plot of ARS (aggregated from PCC, SSIM, COSSIM, RMSE, and JSD; see Methods) illustrating the cell-type composition prediction performance on the regular simulated ST dataset 7. (**B**) PCC, SSIM, COSSIM, RMSE and JSD, as well as the bar plot of ARS illustrating the cell-type composition prediction performance on the regular simulated ST dataset 11. (**C**) The bar plots of Rank PCC, Rank SSIM, Rank COSSIM, Rank RMSE, Rank JSD and ARS summarizing the performance on all of the 20 regular simulated ST datasets.

Generally speaking, SpatialcoGCN show better performance on the majority of datasets. We here take dataset 7 and dataset 11 as examples. The dataset 7 with 1000 spots was simulated by a scRNA-seq data with 1040 cells corresponding to 10 cell types. The results show that SpatialcoGCN achieves the lowest RMSE values (mean RMSE = 0.09) and highest PCC values, SSIM values and COSSIM values (mean PCC = 0.83, mean SSIM = 0.77, mean COSSIM = 0.87; Figure 2A). Finally, SpatialcoGCN gets the highest average rank score (0.96), followed by Cell2location (0.84). The dataset 11 with 1000 spots was simulated by a scRNA-seq data with 10 000 cells corresponding to six cell types. The results on dataset 11 show that SpatialcoGCN has the lowest RMSE values (mean RMSE = 0.09) and highest PCC values, SSIM values and COSSIM values (mean PCC = 0.85, mean SSIM = 0.82, mean COSSIM = 0.92; Figure 2B). On dataset 11, SpatialcoGCN gets the highest average rank score (0.96), followed by RCTD (0.91).

Since the result from single dataset is not so convincing to suggest the better performance of SpatialcoGCN, we further aggregated the overall performance of the methods across all 20 simulated datasets via rank score among the deconvolution methods. Notably, SpatialcoGCN also achieved the highest average PCC rank score, SSIM rank score, COSSIM rank score and lowest RMSE rank score, and its average PCC rank score, SSIM rank score and COSSIM rank score far exceed those of the second method, indicating that it could substantially outperform several state-of-the-arts deconvolution methods (Figure 2C).

### SpatialcoGCN-Sim simulates ST data with awareness of spatial topology

**Figure 3:**
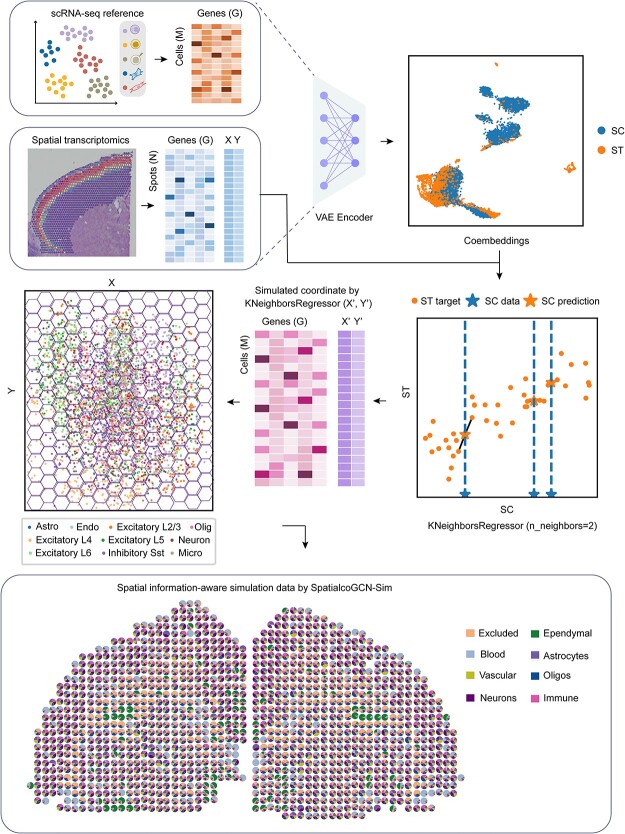
Schematic overview of SpatialcoGCN-Sim, a spatial information-aware ST data simulation method. First, the scRNA-seq data and ST data were projected into a co-embedding space with the aid of the VAE. Subsequently, KNeighborsRegressor was applied in the low-dimensional embedding space to predict the 2D coordinates of the cell based on the coordinates of the spots. To reduce the noise, hexagonal grid representation was employed to represent low-spatial-resolution spots and the center of the hexagon was selected as the coordinates of the spots. Finally, the spatial information–aware simulation data were generated by averaging and normalizing the gene expression of each spot.

Based on a similar deep graph co-embedding framework, SpatialcoGCN-Sim can simulate ST datasets that mimic the ground truth of the proportion of various types of cells on each spot. Unlike previous ST data simulation algorithms used by RCTD [12] and Stereoscope [14], our methodology is based on spot coordinates, resulting in the generation of spatial information-aware ST data that specifies 2D coordinates for every spot. The workflow of SpatialcoGCN-Sim is depicted in Figure 3. Specifically, we used the VAE to map the gene expression in each cell and spot into the low-dimensional embedding space to derive the latent representation. We employed KNN to identify the *k*-nearest spots in reference ST data for each cell in the low-dimension space, then predicted the 2D coordinates of the cell based on the coordinates of the *k* spots. As a result, each cell is assigned unique 2D coordinates. Finally, we used the hexagonal grid to blur the spatial expression pattern, mimicking the low-spatial-resolution spots in ST data. The radius of the spots, in other words, the spatial resolution of the simulated datasets can be customized by adjusting the size of the hexagonal grid.

**Figure 4:**
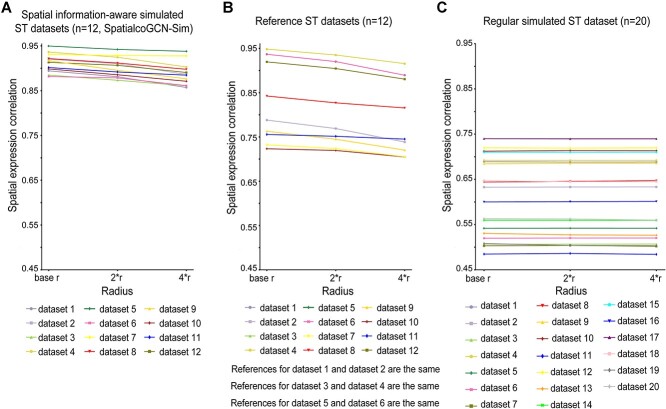
Comparison of spatial expression correlation of different simulated ST datasets and real ST datasets. (**A**) The spatial expression correlation of 12 spatial information-aware simulated ST datasets generated by SpatialcoGCN-Sim. (**B**) The spatial expression correlation of 12 reference ST datasets. (**C**) The spatial expression correlation of 20 regular simulated ST datasets.

To quantitatively evaluate the performance of SpatialcoGCN-Sim, we calculated and compared the spatial expression correlation of spatial information–aware simulated ST datasets (generated by SpatialcoGCN-Sim), reference ST datasets and regular simulated ST datasets (used by RCTD and Stereoscope). We used the coverage range per spot in the ST data as the base radius *r* and consider increasing folds of base radius (i.e. *r*, *2r* and *4r*) as the radius range for spatial expression correlation calculation. The results showed that ST datasets simulated by SpatialcoGCN-Sim show spatial expression correlation levels comparable to the reference ST datasets, which are significantly higher than those of the regular simulated ST datasets (Figure 4). Moreover, on the spatial information–aware simulated ST datasets and reference ST datasets, we observed that the spatial expression correlation decreased when the radius range became wider and more distal spots were taken into consideration. However, regular simulated ST datasets did not show such decay of correlation in response to the distance to the central spot. This result shows SpatialcoGCN-Sim is superior to the regular ST data simulation method as the simulated ST datasets generated by SpatialcoGCN-Sim not only includes the proportion of diverse cell types at each spot but also captures the implicated spatial pattern. Therefore, SpatialcoGCN-Sim would provide more realistic simulation data for model training and evaluation with more enriched spatial information.

### Performance of SpatialcoGCN on the spatial information–aware simulated ST datasets

**Figure 5:**
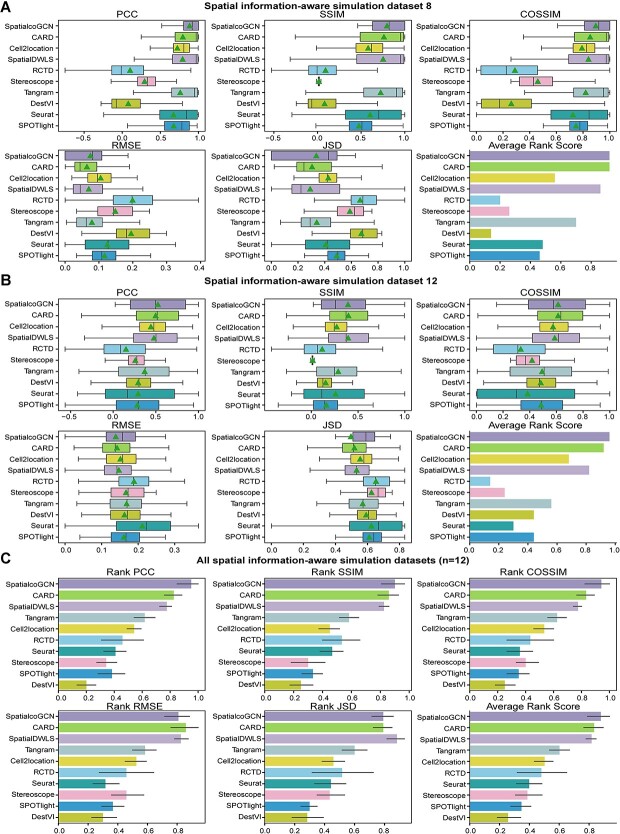
Evaluation of deconvolution performance on spatial information–aware simulated datasets. Comparing the performance of SpatialcoGCN against nine other methods with the capability of deconvoluting cell types for each ST spot, we present our results as boxplots and bar plots. Boxplots: center line, median; box limits, upper and lower quartiles; triangle: mean. Bar plots: data are presented as mean values ± 90% confidence intervals. (**A**) The boxplots of PCC, SSIM, COSSIM, RMSE and JSD, as well as the bar plot of ARS (aggregated from PCC, SSIM, COSSIM, RMSE and JSD; see Methods) illustrating the cell type composition prediction performance on the spatial information–aware simulated ST dataset 8. (**B**) PCC, SSIM, COSSIM, RMSE and JSD, as well as the bar plot of ARS illustrating the cell-type composition prediction performance on the spatial information–aware simulated ST dataset 12. (**C**) The bar plots of Rank PCC, Rank SSIM, Rank COSSIM, Rank RMSE, Rank JSD and ARS summarizing the performance on all of the 12 spatial information–aware simulated ST datasets.

The regular simulated dataset without specific awareness of spatial pattern may underestimate the performance of deconvolution methods that emphasize spatial continuity and patterning, such as CARD [9]. To this end, we further evaluated the deconvolution methods on 12 spatial information–aware simulated ST datasets. These datasets were generated by integrating eight scRNA-seq datasets with nine real ST datasets from the same region or tissue type (Supplementary Table 1). The performance of SpatialcoGCN and the nine popular deconvolutions methods is reported (Figure 5). For example, dataset 8 has 953 spots, with each spot containing 4–15 cells. It was generated by an scRNA-seq data (Smart-seq; mouse cortex) with 14 249 cells corresponding to 15 cell types and a real ST data (seqFISH+; mouse cortex) with 524 spots. The results show that SpatialcoGCN and CARD achieve the highest average rank score (0.92), followed by SpatialDWLS [11] (0.86). SpatialcoGCN also achieves the highest PCC values, SSIM values and COSSIM values (mean PCC = 0.88, mean SSIM = 0.80, mean COSSIM = 0.89; Figure 5A). A similar result was observed in dataset 12, which contains 1179 spots. Dataset 12 was generated by an scRNA-seq data (Smart-seq; mouse cortex) with 13 823 cells corresponding to 15 cell types and a real ST data (seqFISH+; mouse cortex) with 1154 spots. In the prediction results for all cell types of datasets 12, SpatialcoGCN showed the highest average rank score (0.96), followed by CARD (0.92). SpatialcoGCN also exhibited the highest PCC values and COSSIM values (mean PCC = 0.52, mean COSSIM = 0.61, Figure 5B). Meanwhile, SpatialcoGCN had the lowest RMSE values and JSD values (mean RMSE = 0.14, mean JSD = 0.49; Figure 5B).

To comprehensively quantify the performance of the deconvolution methods, we again aggerated the performance across 12 spatial information–aware simulated ST datasets via rank score. The result is summarized in Figure 5C. Briefly, SpatialcoGCN outperformed the other methods in terms of PCC, SSIM and COSSIM. Meanwhile, SpatialcoGCN ranked third in terms of RMSE and JSD metrics, with inferior rank score than CARD and SpatialDWLS. Nevertheless, the differences in RMSE and JSD metrics is not so prominent and the aggregated ARS, which summarizes all the other five performance evaluation metrics, demonstrates that SpatialcoGCN still ranks best in overall performance, followed by CARD (Figure 5C).

In the above benchmarking, we assumed that the gene expression in each spot was only determined by the mixture of cells within the spot. However, in some ST experiments, such as 10X Visium, the RNAs may diffuse to neighboring spots during the permeabilization step. We tested the effect of noise on the performance of SpatialcoGCN on two sets of datasets with various noise addition configurations (see Methods for details). On these noisy datasets, the mean performance of SpatialcoGCN remains consistently the same, although the variation moderately increased as the degree of noise increased (Supplementary Figure 5). These results suggest that SpatialcoGCN was robust to perturbation from the neighbor spots.

### Evaluation on blurred high-resolution ST datasets

**Figure 6:**
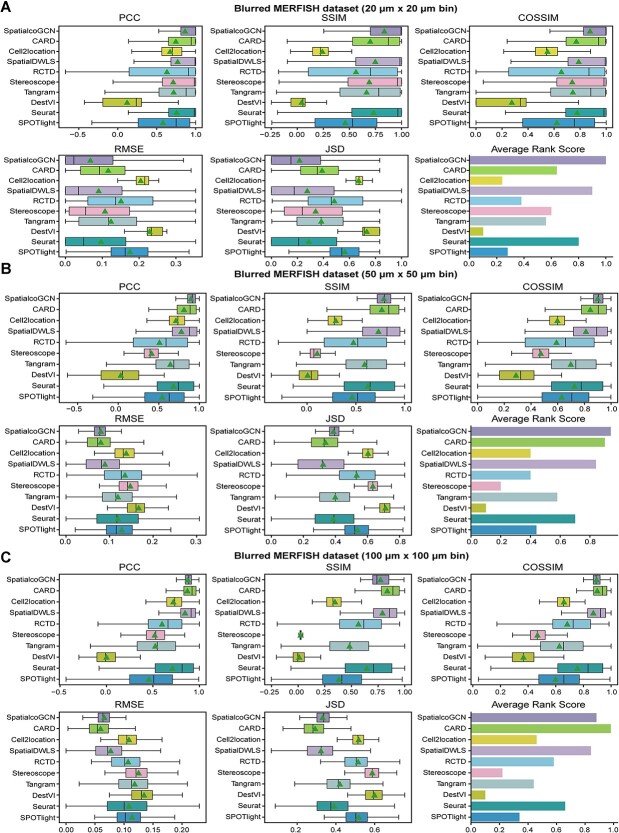
Evaluation of deconvolution performance in the blurred MERFISH datasets at different resolutions. Comparing the performance of SpatialcoGCN against nine other methods with the capability of deconvoluting cell types for each ST spot, we present our results as boxplots and bar plots. Boxplots: center line, median; box limits, upper and lower quartiles; triangle: mean. Bar plots: data are presented as mean values ± 90% confidence intervals. (**A**) The boxplots of PCC, SSIM, COSSIM, RMSE and JSD, along with the bar plot for ARS (aggregated from PCC, SSIM, COSSIM, RMSE and JSD, see Methods) illustrating the cell-type composition prediction performance on the blurred MERFISH dataset at 20 μm resolution (i.e. blurred with 20 μm × 20 μm bin, see Methods). (**B**) The boxplots of PCC, SSIM, COSSIM, RMSE and JSD, along with the bar plot for ARS illustrating the cell-type composition prediction performance on the blurred MERFISH dataset at 50 μm resolution. (**C**) The boxplots of PCC, SSIM, COSSIM, RMSE and JSD, along with the bar plot for ARS illustrating the cell-type composition prediction performance on the blurred MERFISH dataset at 100 μm resolution.

The above assessment relied on simulated data. With the advances of ST techniques, cell resolution platforms like MERFISH and Stereo-seq provide new opportunities for benchmarking the deconvolution method on a real dataset. By blurring such high-resolution dataset with a certain size of binning, the low-resolution spot can be obtained where its ground truth is naturally the raw, high-resolution data. We first generated blurred MERFISH datasets using three different binning sizes of 20, 50 and 100 μm (see Methods for details). Intuitively, datasets with lower resolution are more challenging to deconvolute. Indeed, as the resolution of the dataset drops, the performances of most methods decrease modestly (Figure 6), and such a tendency was most evident for Tangram [15]. However, one exception is CARD [9], which ranks the best in the 100 μm resolution dataset but shows worse ranking in 20 and 50 μm resolutions, indicating this method has been fine-tuned to solve low-resolution data. On the other hand, SpatialcoGCN and SpatialDWLS [11] steadily rank top three for all of the resolutions, and SpatialcoGCN ranks the best in both 20 and 50 μm resolutions (Figure 6).

In addition, we utilized the Stereo-seq mouse brain dataset from Chen *et al*. [28] (Supplementary Figure 6A) to verify SpatialcoGCN. The blurred Stereo-seq data were generated with a similar method to the blurred MERFISH datasets and the binning size of 100 × 100 coordinate unit (bin100). The results demonstrate that SpatialcoGCN, followed by CARD, had the best capability to accurately re-capture the cell composition in each bin (Supplementary Figure 6B). Taken together, these results demonstrate the competitive performance of SpatialcoGCN in the real data-derived benchmarking.

### SpatialcoGCN identifies tumor area and tumor subclones in human DCIS data

**Figure 7:**
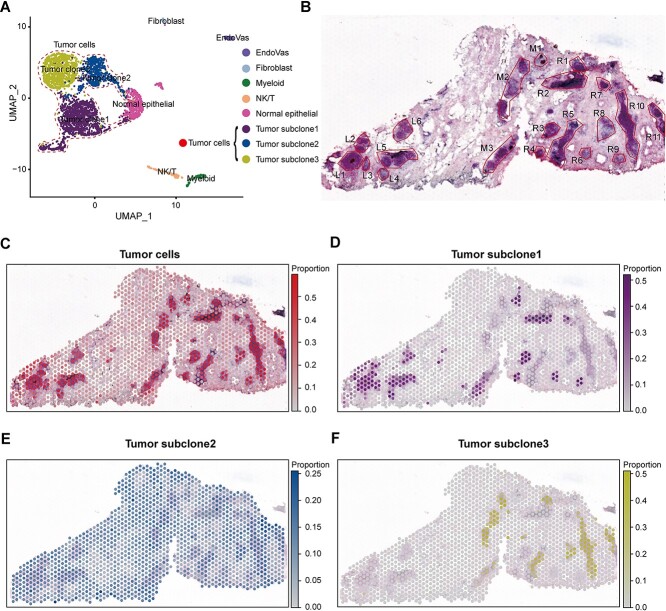
Visualization of SpatialcoGCN’s deconvolution results on DCIS datasets. (**A**) UMAP of DCIS scRNA-seq reference, showing the six (five non-tumor cell types plus one tumor cell) or the eight (five non-tumor cell types plus three tumor subclones) cell type categorization. (**B**) H&E image of the DCIS tissue section with annotated ductal tumor regions. (**C**) The spatial distribution of tumor cells estimated by SpatialcoGCN. (**D**) The spatial distribution of tumor subclone1 estimated by SpatialcoGCN. (**E**) The spatial distribution of tumor subclone2 estimated by SpatialcoGCN. (**F**) The spatial distribution of tumor subclone3 estimated by SpatialcoGCN.

**Figure 8:**
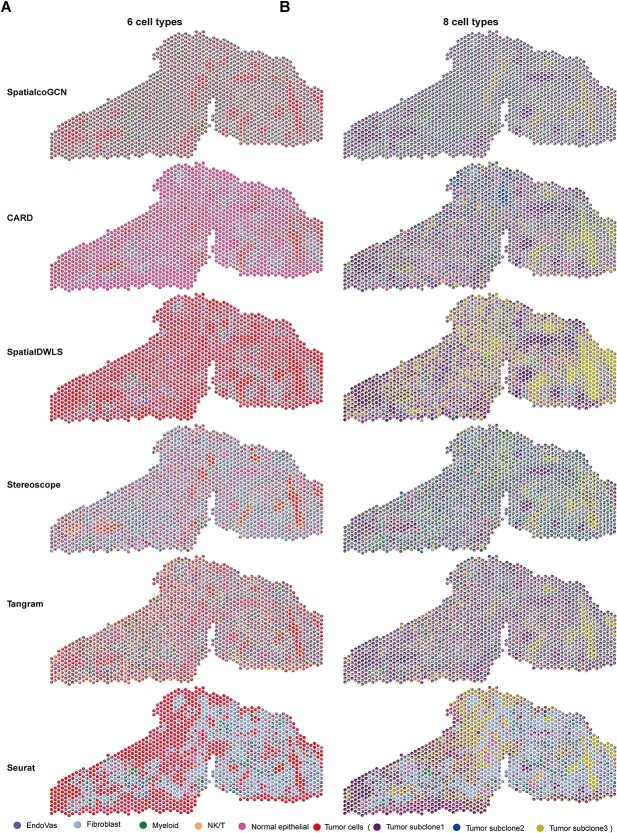
Comparison of deconvolution results by different methods on DCIS datasets. The spatial distributions of cell type proportion predicted by SpatialcoGCN and other five deconvolution methods using (**A**) six cell types scRNA-seq reference and (**B**) eight cell types scRNA-seq reference, respectively, are shown. Each pie represents the cell type proportions in each spot in the ST slide, and colors represent different cell types.

In the benchmarking on the simulation datasets, we evaluated the performance of SpatialcoGCN on scRNA-seq and ST datasets with varying numbers of cell types (from 6 to 54; Supplementary Tables 1 and 2), demonstrating its robustness to different numbers of cell types. However, the definition of cell types, especially subtypes, could be ambiguous in a real dataset. Tumor cells and their subclones constitute typical samples for such cases. Here, we tested the robustness of SpatialcoGCN to different cell type annotations based on the DCIS dataset. The scRNA-seq data from the DCIS dataset have two different sets of cell type annotations: six cell types (five non-tumor cell types plus tumor cells) and eight cell types (the tumor cells were categorized into three subclones, see Methods for more details; Figure 7A). As for the DCIS ST data, 20 ductal tumor regions have been labeled on histopathology (Figure 7B). The results demonstrate that SpatialcoGCN could accurately identify the tumor and subclone regions. We observed that most tumor cells were located in the DCIS regions on the hematoxylin and eosin (H&E) slide (Figure 7C). Moreover, different tumor subclones exhibited distinct spatial patterns, reflecting extensive intratumor heterogeneity [32]. Specifically, subclone2 was dispersed in many ductal areas but had a low proportion; subclone3 was mainly concentrated on the right (R) ducts; subclone1 was presented in both left and right (R) ductal regions but exhibited a well-clustered distribution (Figure 7D–F). Based on the visualization of DCIS, we found that SpatialcoGCN, CARD and Stereoscope [14] had the capability to accurately locate tumors and subclones to spatial locations (Figure 8A and B, Supplementary Figure 7A and B), while some other methods are confused by the heterogeneity of the tumor subclones and could not accurately to locate tumors in the ST-transcriptome map. For non-tumor cell types, SpatialcoGCN, SpatialDWLS, Stereoscope and Seurat [16] also showed similar patterns of cell type distribution no matter whether six or eight cell type annotations were used (taking NK/T cells as an example, Supplementary Figure 7C–F). We also noticed that some immune cells, such as T cells and NK cells, were more enriched in the areas adjacent to the ducts, especially L4, M1, M2, R1, R5 and R10 (Figure 7B, Supplementary Figure 7C). These results indicated that SpatialcoGCN could capture the spatial heterogeneity of cell types without being significantly affected by the ambiguous cell type annotation.

### SpatialcoGCN detects the location of cell types during the development of human heart using either internal or external reference

According to the previous benchmarking study [19], in the deconvolution analysis, two types of references could be considered: (1) internal reference, i.e. using the matched single-cell resolution ST dataset itself as the reference, and (2) external reference, i.e. using other scRNA-seq datasets from the same tissue as the reference. Recently, a spatiotemporal atlas [30] of the human developing heart [4.5–5, 6.5 and 9 PCW] was established by utilizing ST technology, amalgamating scRNA-seq and ISS [30]. This atlas provides a unique opportunity to compare the deconvolution results based on either internal (using single-cell-level ISS ST data as the reference) and external (using scRNA-seq data from similar biological samples as the reference) references.

**Figure 9:**
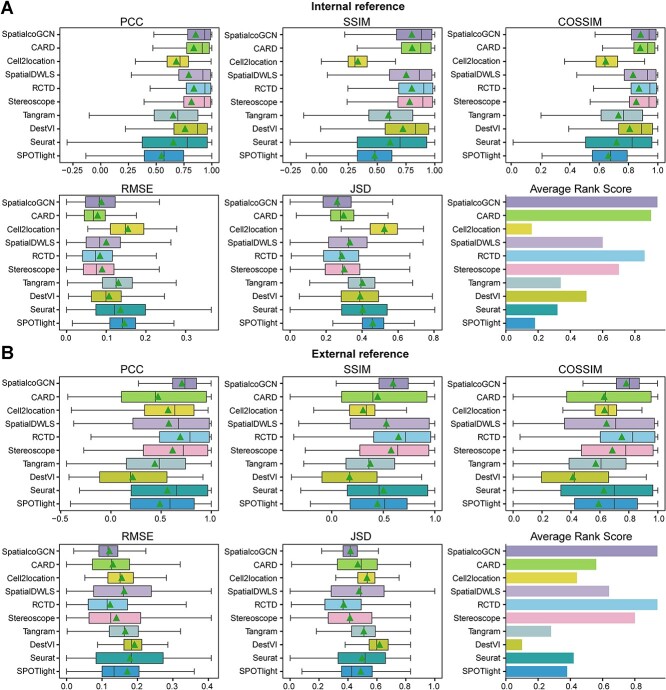
Performance of SpatialcoGCN on developing human heart ISS data. The boxplots of PCC, SSIM, COSSIM, RMSE and JSD, along with the bar plot for ARS illustrating the cell type composition prediction performance on the developing human heart ISS data using the (**A**) internal reference and (**B**) external reference, are shown.

**Figure 10:**
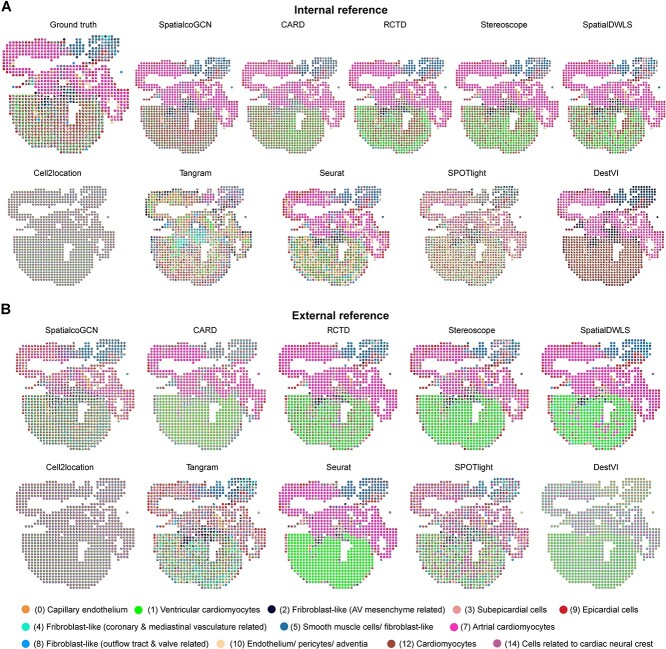
Visualization of SpatialcoGCN’s deconvolution results on developing human heart ISS data. The spatial distributions of cell type proportion estimated for developing human-heart-tissue ISS data using (**A**) internal reference and (**B**) external reference, are shown.

Therefore, we further applied SpatialcoGCN and other deconvolution method on this spatial atlas of the human developing heart. Firstly, the cells in the heart ISS dataset were pooled into pseudo-spots (each of size 454 × 424 square pixels according to their spatial coordinates). We used the internal reference and the external reference, respectively, to deconvolve these pseudo-spots. From evaluate metrics and visualization results (Figures 9 and 10), SpatialcoGCN showed the best performance compared to other methods. More specifically, when using the internal reference, SpatialcoGCN, CARD [9] and RCTD [12] showed superior performance (Figure 9A). The visualization of the deconvolution result demonstrates that atrial cardiomyocytes and ventricular cardiomyocytes were successfully mapped to the atria and ventricular body, respectively (Figure 10A). Furthermore, smooth muscle cells were also correctly mapped to the outflow tract and epicardial cells to the thin outer layer of the heart (Figure 10A), showing good agreement with annotations from the Cell Atlas and previous studies [30, 33]. When the external reference was employed, SpatialcoGCN, RCTD and Stereoscope were capable of capturing the expected spatial distribution of cell types and showed superior performance (Figures 9B and 10B). In contrast to results using the internal reference, CARD and DestVI [13] showed the most obvious performance decrease, while the results of Cell2location were significantly improved when using the external reference (Figure 9A and B).

We also try to deconvolute the spots ST based on the external reference. Similar to the strategy used by STRIDE [34], we utilized the scRNA-seq data collected from the heart at 6.5–7 PCW as the reference for deconvolution models and used the ISS cell type map from the original study as the ground truth (Supplementary Figure 8). Because the ST spots cannot precisely align with the ISS map, instead of using quantitative evaluation metrics, we mainly assessed the performance based on the visualization results to check if the result is in line with the spatial pattern suggested by the ISS map. We performed cell-type deconvolution on all samples from three developmental stages (4.5–5, 6.5 and 9 PCW) and mapped all the cell types to the spatial locations (Supplementary Figure 8A–C). Using sample 1 at 4.5–5 PCW, sample 4 at 6.5 PCW and sample 2 at 9 PCW as the exemplary cases, we demonstrated the distribution of distinct cell types across the hierarchical structure of the human heart (Supplementary Figure 8D–J). By analyzing the proportion of cells in each spot, we found that the abundance of ventricular cardiomyocytes dramatically increases during development, even with fewer cells in the spots. However, the abundance of atrial cardiomyocytes did not follow this trend, perhaps coinciding the previous observation that atrium compartments do not expand as dramatically as the ventricle compartments [11]. As expected, the proportion values of different cell types in various locations revealed that ventricular and atrial cardiomyocytes exhibited the highest proportions in the ventricular body and the atria [35], respectively (Supplementary Figure 8E–J). Smooth muscle cells were primarily located in the outflow tract, consistent with the ISS cell type map (Supplementary Figure 8D and G) and in agreement with their expected location [33]. Epicardial cells were accurately mapped to the outer layer of the heart, known as the epicardium. This cell type predominantly exhibited high proportions in spots covering the edges of the heart, in line with its *in vivo* distribution [30] (Supplementary Figure 8H). Adjacent to the epicardial cells on the inner side of the heart, a slightly thicker layer of epicardium-derived cells was observed (Supplementary Figure 8I). Erythrocytes were visible within the hollow cavities, the same areas as they reside *in vivo* [30] (Supplementary Figure 8J). Together, the cell-type mapping performed by SpatialcoGCN was highly consistent with the spatial cell-type map identified in the original study through the integration of ISS and scRNA-seq data (Supplementary Figure 8). Using sample 4 at 6.5 PCW and sample 2 at 9 PCW as the exemplary cases, we further compared the performance of 10 deconvolution methods. SpatialcoGCN, CARD, RCTD, Stereoscope and SpatialDWLS showed superior performance, where the atrial cardiomyocytes and ventricular cardiomyocytes were successfully mapped to the atria and ventricular body, respectively (Supplementary Figure 9).

### SpatialcoGCN discerns cell-type composition pattern on mouse brain dataset

**Figure 11:**
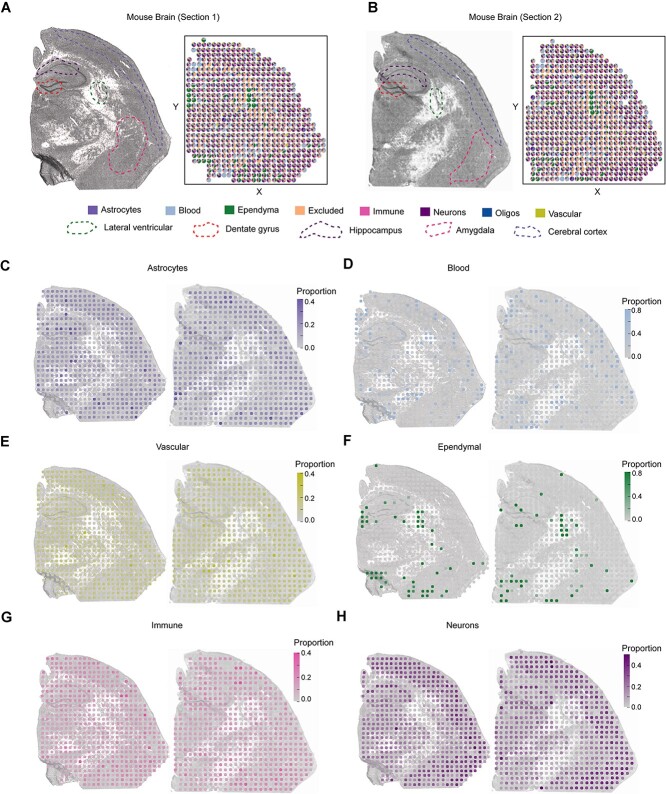
Deconvolution result of SpatialcoGCN on the mouse brain dataset. (**A**, **B**) Spatial scatter pie plot representing the proportions of the cells from the reference atlas within the two captured sections in the adult mouse brain. Two different sections used are: ST1 (ST array, 100 μm spots) and ST2 (ST array, 100 μm spots). The matched histological images are shown, on which the featured brain regions are annotated. (**C**–**H**) The spatial distributions of cell type proportion predicted by SpatialcoGCN for each cell type and on each section.

In the above two real ST datasets, the spatial pattern of (at least part of) the cell types is relatively obvious and well clustered. However, in some tissues like brain, such distribution is often more complicated. Therefore, we also analyzed two 100 micron array ST sections of the mouse brain by the deconvolution methods, using the external hippocampus scRNA-seq that contained a total of 29 519 cells as the reference [14]. The proportions of various cell types per ST spot predicted by SpatialcoGCN are summarized in Figure 11A and B. Further comparison with other deconvolution methods suggests that SpatialcoGCN and Tangram [15] showed superior performance on the mouse brain deconvolution (Supplementary Figure 10). We also note that the spatial organization deduced by SpatialcoGCN is further supported by previous knowledge regarding the distribution of these cell types *in vivo* (Figure 11C–H). For example, astrocytes, the most abundant type of glial cells in the central nervous system, are widely distributed in the mouse brain [31] (Figure 11C). Blood cells are typically distributed in the blood vessels of mouse brains, mainly residing in the same areas [36] (Figure 11D and E). Ependymal cells also align to the ventricular system, forming an epithelial sheet known as the ependyma; thus, observing strong signals for this cell type in the lateral ventricular region is affirmative [37] (Figure 11F). Immune cells were distributed in various brain regions of the mouse, such as the cortex, hippocampus and amygdala, but the expression levels of different brain regions and cells varied prominently [38] (Figure 11G). Neurons populate in regions such as the dentate gyrus, amygdala, cerebral cortex and parts of Ammon’s horn in the hippocampus (Figure 11H), again consistent with the previous findings [37]. These results confirm the accuracy and reliability of SpatialcoGCN on real ST datasets with a complicated spatial pattern.

### SpatialcoGCN can recover the undetected genes in enhancement of ST

ST technologies are limited by either the small number of genes or the low gene detection sensitivity. Since SpatialcoGCN has learned a mapping matrix between scRNA-seq data and ST data, predicting spatial distribution of RNA transcripts has also become feasible for SpatialcoGCN. We used 10-fold cross-validation (see Methods for more details) on five matched datasets (Supplementary Table 3) to evaluate the accuracy of SpatialcoGCN in predicting the spatial distribution of RNA transcripts. Seven methods that have the function of recovering the undetected genes were considered in the performance comparison, namely, Tangram [15], SpaGE [39], Seurat [16], SpaOTsc [40], novoSpaRc [41], LIGER [42] and stPlus [43]. The transcript distribution prediction of SpatialcoGCN was compared against that of each method. Similar to cell type deconvolution, we quantified the prediction performance of each comparable method by calculating the PCC, SSIM, COSSIM, RMSE and JSD between the expression values of a gene in the ground truth observed in the ST dataset and the predicted expression values of the same gene. Again, ARS was used to aggregate the five metrics.

**Figure 12:**
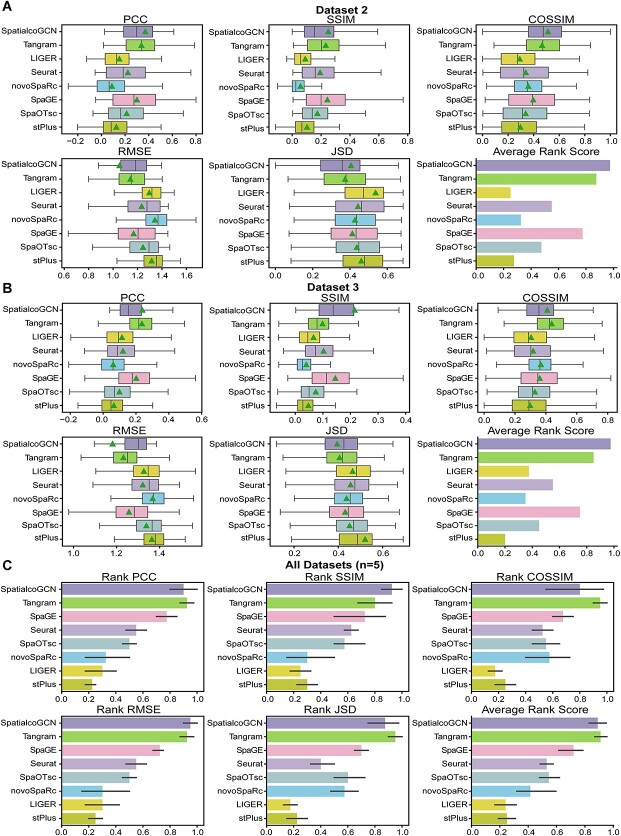
Evaluation of the performance of SpatialcoGCN for recovering undetected genes on ST datasets. The performance of SpatialcoGCN is compared with seven other methods that are capable of predicting the spatial distribution of RNA transcripts. Boxplots: center line, median; box limits, upper and lower quartiles; triangle: mean. Bar plots: data are presented as mean values ± 90% confidence intervals. (**A**) The boxplots of PCC, SSIM, COSSIM, RMSE and JSD and the bar plot of ARS illustrating the performance of each method in predicting the spatial distribution of transcripts in dataset 2. (**B**) The boxplots of PCC, SSIM, COSSIM, RMSE, and JSD and the bar plot of ARS illustrating the performance of each method in predicting the spatial distribution of transcripts in dataset 3. (**C**) The bar plots of Rank PCC, Rank SSIM, Rank COSSIM, Rank RMSE, Rank JSD and ARS summarizing the performance on all of the five matched datasets.

We first demonstrate the comparison result on the dataset 2 that contains 2376 spots and 250 genes. In the 10-fold cross-validation test, each time, 225 genes are used as the training set, and the other 25 genes are used as the test set. The results on this dataset show that SpatialcoGCN achieves the lowest RMSE values (mean RMSE = 1.05) and highest PCC values, SSIM values and COSSIM values (mean PCC = 0.37, mean SSIM = 0.25, mean COSSIM = 0.51, Figure 12A). Notably, SpatialcoGCN gets the highest ARS (0.98), followed by Tangram (0.88). The results on dataset 3 showed similar trends. On this dataset, SpatialcoGCN achieved the lowest RMSE values and JSD values (mean RMSE = 1.18, mean JSD = 0.39) and highest PCC values and SSIM values (mean PCC = 0.24, mean SSIM = 0.22, Figure 12B). SpatialcoGCN also obtained the highest ARS (0.98), followed by Tangram (0.85).

When we compared the overall performance of the methods across all five matched datasets, the results demonstrated that both Tangram and SpatialcoGCN could show superior performance than the other methods (Figure 12C). Note that in all of the tests in recovering the undetected genes, only the ‘not-well-aligned’ external references (as per the definition from the previous study [19]) from the matched scRNA-seq datasets were used, enabling a more realistic benchmarking. Besides, SpatialcoGCN outperformed the other methods in the SSIM rank score and RMSE rank score and ranked second for the other evaluation metrics (Figure 12C). The above results indicate that SpatialcoGCN has at least competitive performance compared to the state-of-the-art methods in predicting the spatial distribution of unseen transcripts in ST data.

### SpatialcoGCN has the proxy to incorporate reference-free deconvolution

Like most deconvolution methods, SpatialcoGCN requires scRNA-seq data as the reference to calculate expression profiles for each cell type. A reference-free deconvolution approach like STdeconvolve [44] provides an alternative strategy for deconvolving cell types when an appropriate reference is not available. STdeconvolve was inspired by the notion of discovering latent topics in collections of documents, which is a common task in natural language processing, and used the latent Dirichlet allocation (LDA) algorithm to infer the putative transcriptional profile for each cell type and the proportional representation of each cell type in each ST spot. Exploiting putative transcriptional profiles enables the incorporation of SpatialcoGCN with STdeconvolve. In STdeconvolve, the number of cell types can be predicted or specified. We tested the performance of the incorporation of SpatialcoGCN and STdeconvolve in both cases.

Using the STdeconvolve results of putative transcriptional profile for each cell type as the reference for SpatialcoGCN, reference-based SpatialcoGCN method and reference-free STdeconvolve can be used in combinatorial fashion. However, the simulated datasets 7 and 11 consisted of 10 and 6 distinct cell types, respectively, while STdeconvolve estimated 12 and 8 cell types, respectively. To align STdeconvolute-predicted cell types to the ground-truth cell types, each predicted cell type was first matched with the ground-truth cell type that had the highest Pearson’s correlation between their transcriptional profiles. The unaligned cell types from STdeconvolute were discarded.

After the alignment of cell types, SpatialcoGCN can be applied using the predicted cell type references from STdeconvolute. The results showed that the performance of the incorporation of SpatialcoGCN and STdeconvolve was better than using STdeconvolve alone, but the improvement is not prominent. Besides, combining SpatialcoGCN and STdeconvolve does not show better performance than using SpatialcoGCN alone (Supplementary Figure 11). Therefore, at the current stage, such combination looks not so feasible. Nonetheless, since there is a prospect to incorporate reference-free deconvolution methods, when the accuracy of predicting cell-type gene expression profiles increases in the future, the performance of SpatialcoGCN combined with reference-free deconvolution would also improve accordingly.

## DISCUSSION

In this study, we proposed SpatialcoGCN, a self-supervised graph-based deep learning model designed to deconvolute ST data. Our approach leverages scRNA-seq data to reveal cellular mixtures present in ST data. We have demonstrated the benefits of SpatialcoGCN on a series of simulated ST data and real ST datasets from human DCIS, developing human heart and mouse brain. The results reveal that our model accurately estimated cell-type proportions and outperformed existing methods in terms of sensitivity–specificity balance and robustness to the impacts from addition of background noise, choice of internal/external reference or alteration in cell type annotations. When applied to real ST data, our method presented results that agreed with previous literature [11, 14, 20, 34]. The superior performance of SpatialcoGCN can be attributed to its ability to leverage scRNA-seq data and the use of a co-graph convolution approach, which helps to capture the spatial relationship between cells and spots in ST data. The learnable mapping matrix between scRNA-seq data and ST data is based on the intrinsic relationship between the two data, and the mapping matrix is dynamically adjusted in the process of minimizing the loss function. It is different from Tangram [15], where the mapping matrix is initialized by a matrix of random numbers drawn from a normal distribution with zero mean and unit standard deviation. According to Tangram, the scRNA-seq data are not directly involved in the initial generation of the mapping matrix. Besides, SpatialcoGCN uses graph convolution to directly infer the possibility of each cell type being in a spot according to the connection weight between the cell and the spot in the link graph, while the connections between cells and spots are not emphasized by Tangram.

There has been one previous method that exploits GCN for ST data deconvolution, i.e. DSTG [26]. Instead of solving the mapping matrix between cell types and spots, DSTG randomly mixes cells from scRNA-seq data to generate pseudo-spots and leverages the similarity between pseudo- and real spots in the CCA-based co-embedding space to build the link graph. A semi-supervised GCN is utilized to minimize a loss function that measures the cross-entropy between the known and the predicted cell compositions of pseudo-spots. The basic assumption of this loss function is that, if the model can accurately predict the cell composition of pseudo-spots, it can properly estimate that of real spots as well. However, the benchmarking results suggest that optimizing the prediction accuracy of pseudo-spot does not always guarantee the global deconvolution performance. Another advantage of solving the mapping matrix instead of pseudo-spot composition in the loss function is that the expression profile of each real-ST spot can be retrieved, enabling the recovery of undetected genes. Besides, SpatialcoGCN incorporates a sophisticated VAE deep learning framework to establish the co-embedding space, which is more robust to the technical noise and batch differences in ST data and scRNA-seq references.

Beyond the deep learning–based method, the idea of finding a non-negative mapping matrix $M$ given scRNA-seq matrix $C$ and ST matrix $S$ is also adopted by statistical methods such as the non-negative least squares (NNLS)–based methods SpatialDWLS [11] and SPOTlight [17]. One main difference between SpatialcoGCN and these NNLS-based methods is that SpatialcoGCN utilizes a deep graph convolution model to represent the links between cells and the spots. Therefore, although not accurate, the main objective of SpatialcoGCN can also be described as running an NNLS-like process of *S* itself and its graph neighbors, on *C* itself and its graph neighbors: which is not the same to directly running NNLS of *S* on *C*. The deep learning optimization method of SpatialcoGCN is more suitable for processing complex models, making full use of the correlation between scRNA-seq data and ST data during the iterative process, while the NNLS optimization algorithm is more suitable for processing linear models. In the deconvolution task, with such a more comprehensive objective and model structure, SpatialcoGCN could outperform NNLS-based methods.

SpatialcoGCN can even predict the spatial distribution of undetected transcripts and achieve commendable performance that is only next to Tangram on benchmarking datasets, although it is not designed for that purpose. Interestingly, the performance in the deconvolution task and that in recovery of undetected transcripts seems somewhat contradictory. A typical example is Tangram: for the task of recovering the undetected genes, Tangram ranks the best; for cell type deconvolution, the ranking of Tangram is within the top 25% ~ 50%, depending on which simulated or real dataset was used. Therefore, to further improve the ability of SpatialcoGCN, the computational framework would be modified to enhance the gene-wise correlation between scRNA-seq and ST data. However, the cost is likely to be a reduction of deconvolution performance. Another future extension of SpatialcoGCN is to map single cells back to their spatial coordinates in tissue, utilizing the specific trait of SpatialcoGCN’s loss function, which will converge to a smaller value when scRNA-seq cells and ST spots match each other. To achieve this purpose, the computational framework should be further modified to fit the links between spots and single cells, rather than the current links between spot and cell types.

Furthermore, we simulated ST data with awareness of spatial topology, taking into account spot coordinates using SpatialcoGCN-Sim. This enabled us to generate data that closely resembled actual data, including the proportions of different types of cells present in each spot. This is an improvement over existing simulation algorithms used in RCTD [12] and Stereoscope [14], which did not include spatial coordinates. As a simulator, SpatialcoGCN-Sim provides flexible, reproducible and diverse simulation. The flexibility of SpatialcoGCN-Sim is demonstrated in two aspects: first, the resolution of simulated data can be adjusted by changing the size of the hexagonal grid; second, SpatialcoGCN-Sim can also simulate ST data from different technical platforms by limiting the number of cells per spot. The simulation result is reproducible; by fixing the random seed, same simulated ST data can be reproduced. Conversely, different random seeds can generate different outcomes from the same ST reference data, reflecting the diversity of the method. Our approach promises to facilitate the development of novel computational methods for ST data analysis.

It should be emphasized that our simulation method directly predicts the location of each cell in scRNA-seq data, which may be helpful for single-cell spatial mapping in diverse tissue types to resolve their spatial organization. With this merit, our simulated ST data can be leveraged not just for cell type deconvolution but also as the gold standard for other type of ST analysis methods that map single cells back to their spatial coordinates in tissue sections, such as CellTrek [29] and CytoSPACE [45]. One converse proposition is that CellTrek would also be used as a method to simulate ST data, just like SpatialcoGCN-Sim (CytoSPACE cannot be used to simulate ST data for deconvolution because CytoSPACE itself requires the deconvolution results as its input). To check this possibility, we applied CellTrek to create spatial coordinates for cells first and then aggregated them into pseudo-spots. We evaluated the average expression correlation between the central point and its neighbors within a certain radius in the simulated datasets (see Methods for the details of spatial expression correlation). The results showed that ST datasets simulated by SpatialcoGCN-Sim show spatial expression correlation levels comparable to the reference ST datasets, which are also significantly higher than the spatial expression correlation levels of the ST datasets simulated by CellTrek (Supplementary Figure 12A). On the other hand, SpatialcoGCN-Sim could generate a more diverse dataset based on one reference. We generated five simulated data using SpatialcoGCN-Sim and CellTrek with the same reference and evaluated the difference in expression profiles between these five simulated data. The results showed that 93.9% of the spots in each two simulation datasets generated by SpatialcoGCN-Sim had different expression profiles on average, with an average difference level of 0.41. However, the simulation data generated by CellTrek only show a marginal difference between each other (Supplementary Figure 12B). Together, these results demonstrate that SpatialcoGCN-Sim could indeed produce a spatial correlation–aware ST dataset with substantial divergence, while CellTrek, which was not designed for ST data simulation, show much less capability in this term. In fact, unlike CellTrek, the spatial mapping in SpatialcoGCN-Sim is not necessarily accurate, a loosely aligned cell-to-spot mapping is enough for a reasonable and flexible generation of spatial information–aware ST simulation data.

In conclusion, SpatialcoGCN is a useful and efficient tool for deconvoluting ST data that enables researchers to map gene expression within the spatial context of tissue with remarkable precision. Our simulation method for ST datasets is a valuable contribution that will enable researchers to generate high-quality simulated ST data for testing and validating various computational methods. We hope our proposed ST data deconvolution and simulation approaches will help advance the field of spatial transcriptomics and create novel avenues for exploring the spatial organization of cells within intricate tissues in the future.

**Key Points**

- SpatialcoGCN is a deep graph co-embedding based method to deconvolute spatial transcriptomics data into cell type composition and recover the undetected genes per spot.
- Benchmarking of SpatialcoGCN on both simulated datasets and real datasets have not only validated its better performance in comparison with state-of-the-arts deconvolution methods, but also highlighted its capability to impute spatial distribution of undetected transcripts in ST data.
- SpatialcoGCN was applied to study the spatial organization of the human ductal carcinoma *in situ*, human developing heart ST dataset and mouse brain dataset, where the cell-type mapping performed by SpatialcoGCN was highly consistent with the spatial cell-type map identified in the original study.
- SpatialcoGCN-Sim is a similar deep graph co-embedding framework, for the spatial information-aware simulation of ST data.
- The simulated ST datasets generated by SpatialcoGCN-Sim grasps the details on each spot, including the number of cells, the proportion of cell types and the source of every single cell in each spot.

## Supplementary Material

Supplementary_Materials_rev2_bbae130

## Data Availability

All simulated datasets used in this study are publicly available at Figshare (https://figshare.com/articles/dataset/SpatialcoGCN_data/22682611). The Stereo-seq mouse brain dataset was obtained from https://db.cngb.org/stomics/mosta/. The MERFISH dataset were obtained from https://datadryad.org/stash/dataset/doi:10.5061/dryad.8t8s248/. The ISS heart datasets are available in the previous reference PMID: 35753702. The mouse brain dataset is available in the previous reference PMID: 33037292. The DCIS dataset is obtained from the previous reference PMID: 35314812. The spot-level human heart was obtained from public database (Mendeley Data; https://data.mendeley.com/datasets/mbvhhf8m62/2). The source code of this article is freely available on GitHub, at https://github.com/wwYinYin/SpatialcoGCN.

## Author Biographies

**Wang Yin** is a PhD candidate at Department of Biomedical Informatics, Peking University. His research interest is spatial transcriptomics data analysis and deep learning. He has also studied at Department of Neurobiology.

**You Wan** is a professor at Department of Neurobiology, School of Basic Medical Sciences, Neuroscience Research Institute, Peking University. His research interest includes the learning and memory, emotion and cognition of pain chronicity and its regulation mechanism.

**Yuan Zhou** is an investigator at Department of Biomedical Informatics, Peking University. His research interest includes transcriptomic and epitranscriptomic bioinformatics.
