# Supplementary material for: SpatialcoGCN: deconvolution and spatial information–aware simulation of spatial transcriptomics data via deep graph co-embedding
Source: Brief Bioinform. 2024 Mar 31;25(3):bbae130. doi: 10.1093/bib/bbae130 (PMC10982953; doi:10.1093/bib/bbae130)
Supplement: Supplementary_Materials_rev2_bbae130 [file supplementary_materials_rev2_bbae130.docx]

**Supplementary Table 1:** 12 pairs of matched scRNA-seq data and real ST data for generating spatial information-aware simulation of ST datasets by SpatialcoGCN-Sim.

| **Dataset ID** |  | **Single cell RNA sequencing (scRNA-seq) Data Summary** | | | | | | |
| --- | --- | --- | --- | --- | --- | --- | --- | --- |
|  |  | **Sample Information** | |  | **scRNA-seq Information** | | | |
|  |  | Species | Tissue |  | Cell number | Gene Number | Cell Type Number | Data Source (the scRNA-seq data, real ST data, and simulated ST data are uploaded to:  https://figshare.com/articles/dataset/SpatialcoGCN_data/22682611) |
| 1 |  | Mouse | Brain |  | 73363 | 45768 | 54 | https://portal.brain-map.org/atlases-and-data/rnaseq/Mouse-whole-cortex-and-hippocampus-smart-seq |
| 2 |  | Mouse | Brain |  | 1169213 | 28540 | 53 | https://portal.brain-map.org/atlases-and-data/rnaseq/Mouse-whole-cortex-and-hippocampus-10x |
| 3 |  | Mouse | Kidney |  | 10146 | 29244 | 8 | https://www.ncbi.nlm.nih.gov/geo/query/acc.cgi?acc=GSE117089 |
| 4 |  | Mouse | Kidney |  | 26654 | 24965 | 8 | https://www.ncbi.nlm.nih.gov/geo/query/acc.cgi?acc=GSE139107 |
| 5 |  | Mouse | Kidney |  | 10146 | 29244 | 8 | https://www.ncbi.nlm.nih.gov/geo/query/acc.cgi?acc=GSE117089 |
| 6 |  | Mouse | Kidney |  | 26654 | 24965 | 8 | https://www.ncbi.nlm.nih.gov/geo/query/acc.cgi?acc=GSE139107 |
| 7 |  | Mouse | Kidney |  | 26654 | 24965 | 8 | https://www.ncbi.nlm.nih.gov/geo/query/acc.cgi?acc=GSE139107 |
| 8 |  | Mouse | Visual cortex |  | 14249 | 34041 | 15 | https://portal.brain-map.org/atlases-and-data/rnaseq/Mouse-v1-and-alm-smart-seq |
| 9 |  | Mouse | Visual cortex |  | 14249 | 34041 | 15 | https://portal.brain-map.org/atlases-and-data/rnaseq/Mouse-v1-and-alm-smart-seq |
| 10 |  | Mouse | Visual cortex |  | 14249 | 34041 | 15 | https://portal.brain-map.org/atlases-and-data/rnaseq/Mouse-v1-and-alm-smart-seq |
| 11 |  | Mouse | Visual cortex |  | 14249 | 34041 | 15 | https://portal.brain-map.org/atlases-and-data/rnaseq/Mouse-v1-and-alm-smart-seq |
| 12 |  | Mouse | Visual cortex |  | 14249 | 34041 | 15 | https://portal.brain-map.org/atlases-and-data/rnaseq/Mouse-v1-and-alm-smart-seq |

(continued)

| **Dataset ID** |  | **Real Spatial Transcriptome (ST) Data Summary** | | | | | |
| --- | --- | --- | --- | --- | --- | --- | --- |
|  |  | **Sample Information** | |  | **ST Information** | | |
|  |  | Species | Tissue |  | Spot Number | Gene Number | Data Source |
| 1 |  | Mouse | Brain |  | 3805 | 27999 | https://www.ncbi.nlm.nih.gov/geo/query/acc.cgi?acc=GSE153424 |
| 2 |  | Mouse | Brain |  | 3805 | 27999 | https://www.ncbi.nlm.nih.gov/geo/query/acc.cgi?acc=GSE153424 |
| 3 |  | Mouse | Kidney |  | 1888 | 31053 | https://www.ncbi.nlm.nih.gov/geo/query/acc.cgi?acc=GSE154107 |
| 4 |  | Mouse | Kidney |  | 1888 | 31053 | https://www.ncbi.nlm.nih.gov/geo/query/acc.cgi?acc=GSE154107 |
| 5 |  | Mouse | Kidney |  | 1835 | 31053 | https://www-ncbi-nlm-nih-gov.ezproxy.u-pec.fr/geo/query/acc.cgi?acc=GSM5224979 |
| 6 |  | Mouse | Kidney |  | 1835 | 31053 | https://www-ncbi-nlm-nih-gov.ezproxy.u-pec.fr/geo/query/acc.cgi?acc=GSM5224979 |
| 7 |  | Mouse | Kidney |  | 2064 | 31053 | https://www-ncbi-nlm-nih-gov.ezproxy.u-pec.fr/geo/query/acc.cgi?acc=GSM5224979 |
| 8 |  | Mouse | Cortex |  | 524 | 10000 | https://github.com/CaiGroup/seqFISH-PLUS |
| 9 |  | Mouse | Visual cortex |  | 1549 | 1020 | https://www.starmapresources.com/data |
| 10 |  | Mouse | Primary visual cortex (VISp) |  | 6000 | 119 | https://github.com/spacetx-spacejam/data |
| 11 |  | Mouse | Primary visual cortex (VISp) |  | 11426 | 80 | https://github.com/spacetx-spacejam/data |
| 12 |  | Mouse | Primary visual cortex (VISp) |  | 1154 | 42 | https://github.com/spacetx-spacejam/data |

**Supplementary Table 2:** Detailed information of 20 regular simulated spatial transcriptomics and scRNA-seq datasets.

| **Dataset ID** | **Species** | **Tissue** | **Simulated ST Spot Number** | **Simulated ST Gene Number** | **Reference scRNA-seq Cell Number** | **Reference scRNA-seq Gene Number** | **Reference scRNA-seq Cell Type Number** | **Reference scRNA-seq Data Source**  **(the reference scRNA-seq data, simulated ST data, and scRNA-seq data used for simulation are uploaded to: https://figshare.com/articles/dataset/SpatialcoGCN_data/22682611)** |
| --- | --- | --- | --- | --- | --- | --- | --- | --- |
| 1 | Human | Liver | 1000 | 19850 | 3821 | 18320 | 6 | https://www.ncbi.nlm.nih.gov/geo/query/acc.cgi?acc=GSE125449 |
| 2 | Human | Liver | 1000 | 26160 | 6948 | 19909 | 6 | https://www.ncbi.nlm.nih.gov/geo/query/acc.cgi?acc=GSE115469 |
| 3 | Human | Lung | 1000 | 38150 | 10000 | 25485 | 13 | https://www.ebi.ac.uk/ena/browser/view/PRJNA591860 |
| 4 | Human | Heart | 1000 | 29484 | 10000 | 17921 | 8 | https://www.ncbi.nlm.nih.gov/geo/query/acc.cgi?acc=GSE156703 |
| 5 | Human | Heart | 1000 | 31580 | 10000 | 17923 | 8 | https://www.ncbi.nlm.nih.gov/geo/query/acc.cgi?acc=GSE156703 |
| 6 | Human | Pancreas | 1000 | 17499 | 2282 | 21198 | 6 | https://cblast.gao-lab.org/Enge/Enge.h5 |
| 7 | Human | Pancreas | 1000 | 17499 | 1040 | 21572 | 10 | https://cblast.gao-lab.org/ALIGNED_Homo_sapiens_Pancreas/ALIGNED_Homo_sapiens_Pancreas.h5 |
| 8 | Human | Pancreas | 1000 | 21198 | 943 | 21413 | 6 | https://cblast.gao-lab.org/ALIGNED_Homo_sapiens_Pancreas/ALIGNED_Homo_sapiens_Pancreas.h5 |
| 9 | Mouse | Pancreas | 1000 | 14860 | 1382 | 19479 | 8 | https://cblast.gao-lab.org/Quake_Smart-seq2/Quake_Smart-seq2.h5 |
| 10 | Mouse | Trachea | 1000 | 18388 | 6937 | 27083 | 6 | https://cblast.gao-lab.org/Plasschaert/Plasschaert.h5 |
| 11 | Human | Liver | 1000 | 18328 | 10000 | 19653 | 6 | https://www.ncbi.nlm.nih.gov/geo/query/acc.cgi?acc=GSE156337 |
| 12 | Human | Liver | 1000 | 20007 | 8785 | 25644 | 6 | https://www.ncbi.nlm.nih.gov/geo/query/acc.cgi?acc=GSE124395 |
| 13 | Human | Lung | 1000 | 25734 | 10000 | 34460 | 13 | https://www.ncbi.nlm.nih.gov/geo/query/acc.cgi?acc=GSE127465 |
| 14 | Human | Lung | 1000 | 25734 | 10000 | 24262 | 9 | https://www.ncbi.nlm.nih.gov/geo/query/acc.cgi?acc=GSE131907 |
| 15 | Human | Kidney | 1000 | 27345 | 10000 | 30831 | 10 | https://www.ncbi.nlm.nih.gov/geo/query/acc.cgi?acc=GSE121862 |
| 16 | Mouse | Kidney | 1000 | 24965 | 10000 | 29191 | 8 | https://www.ncbi.nlm.nih.gov/geo/query/acc.cgi?acc=GSE117089 |
| 17 | Human | Heart | 1000 | 17926 | 10000 | 27663 | 8 | https://www.ebi.ac.uk/ena/browser/view/PRJEB39602 |
| 18 | Human | Pancreas | 1000 | 21198 | 7944 | 17338 | 6 | https://cblast.gao-lab.org/Baron_human/Baron_human.h5 |
| 19 | Human | Pancreas | 1000 | 21625 | 8494 | 17429 | 10 | https://cblast.gao-lab.org/Baron_human/Baron_human.h5 |
| 20 | Human | Pancreas | 1000 | 21625 | 2282 | 21198 | 6 | https://cblast.gao-lab.org/Enge/Enge.h5 |

**Supplementary Table 3:** Detailed information of 5 matched real spatial transcriptomics and real scRNA-seq datasets for evaluation of recovering undetected genes.

| **Dataset ID** |  | **ST Data Summary** | | | | | | | | |
| --- | --- | --- | --- | --- | --- | --- | --- | --- | --- | --- |
|  |  | **Sample Information** | | | | |  | **ST Data Information** | | |
|  |  | Platform | Species | | Tissue | |  | Spot Number | Gene Number | Data Source |
| 1 |  | seqFISH+ | Mouse | | Cortex | |  | 524 | 10000 | https://github.com/CaiGroup/seqFISH-PLUS |
| 2 |  | MERFISH | Mouse | | Primary visual cortex (VISp) | |  | 2399 | 268 | https://github.com/spacetx-spacejam/data/ |
| 3 |  | STARmap | Mouse | | Visual cortex | |  | 1549 | 1020 | https://www.starmapresources.com/data |
| 4 |  | ISS | Mouse | | Primary visual cortex (VISp) | |  | 6000 | 119 | https://github.com/spacetx-spacejam/data |
| 5 |  | ExSeq | Mouse | | Primary visual cortex (VISp) | |  | 1154 | 42 | https://github.com/spacetx-spacejam/data |
| **Dataset ID** |  | **scRNA-seq Data Summary** | | | | | | | | |
|  |  | Cell Number | | Gene Number | | Data Source | | | | |
| 1 |  | 14249 | | 34041 | | https://portal.brain-map.org/atlases-and-data/rnaseq/mouse-v1-and-alm-smart-seq | | | | |
| 2 |  | 14249 | | 34041 | | https://portal.brain-map.org/atlases-and-data/rnaseq/mouse-v1-and-alm-smart-seq | | | | |
| 3 |  | 14249 | | 34041 | | https://portal.brain-map.org/atlases-and-data/rnaseq/mouse-v1-and-alm-smart-seq | | | | |
| 4 |  | 14249 | | 34041 | | https://portal.brain-map.org/atlases-and-data/rnaseq/mouse-v1-and-alm-smart-seq | | | | |
| 5 |  | 14249 | | 34041 | | https://portal.brain-map.org/atlases-and-data/rnaseq/mouse-v1-and-alm-smart-seq | | | | |

**
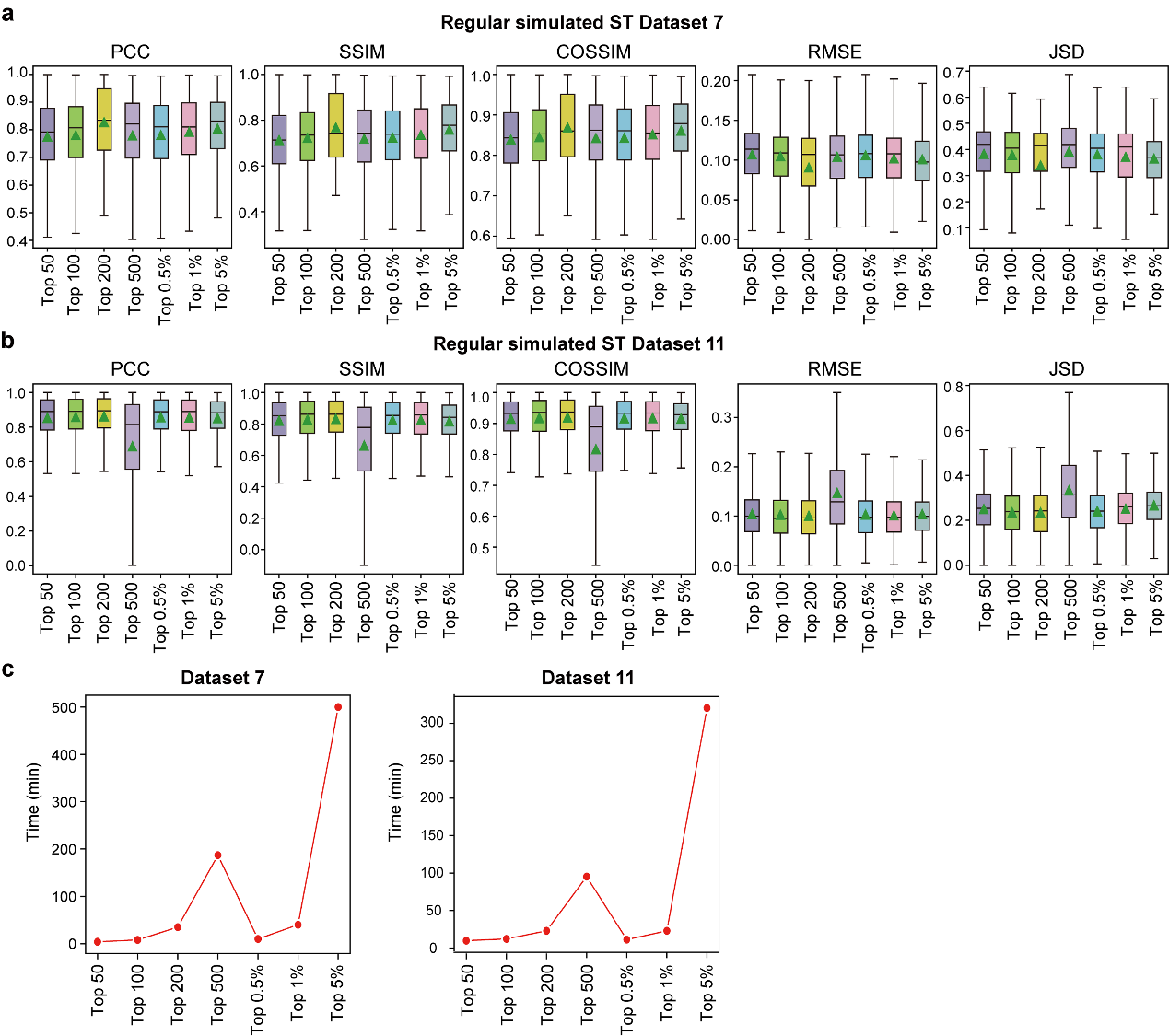
**

**Supplementary Figure 1. The performance of SpatialcoGCN using different marker gene sets.** (**a**) The performance of SpatialcoGCN using different marker gene sets on regular simulated dataset 7. (**b**) The performance of SpatialcoGCN using different marker gene sets on regular simulated dataset 11. (**c**) The computation time of SpatialcoGCN using different marker gene sets.


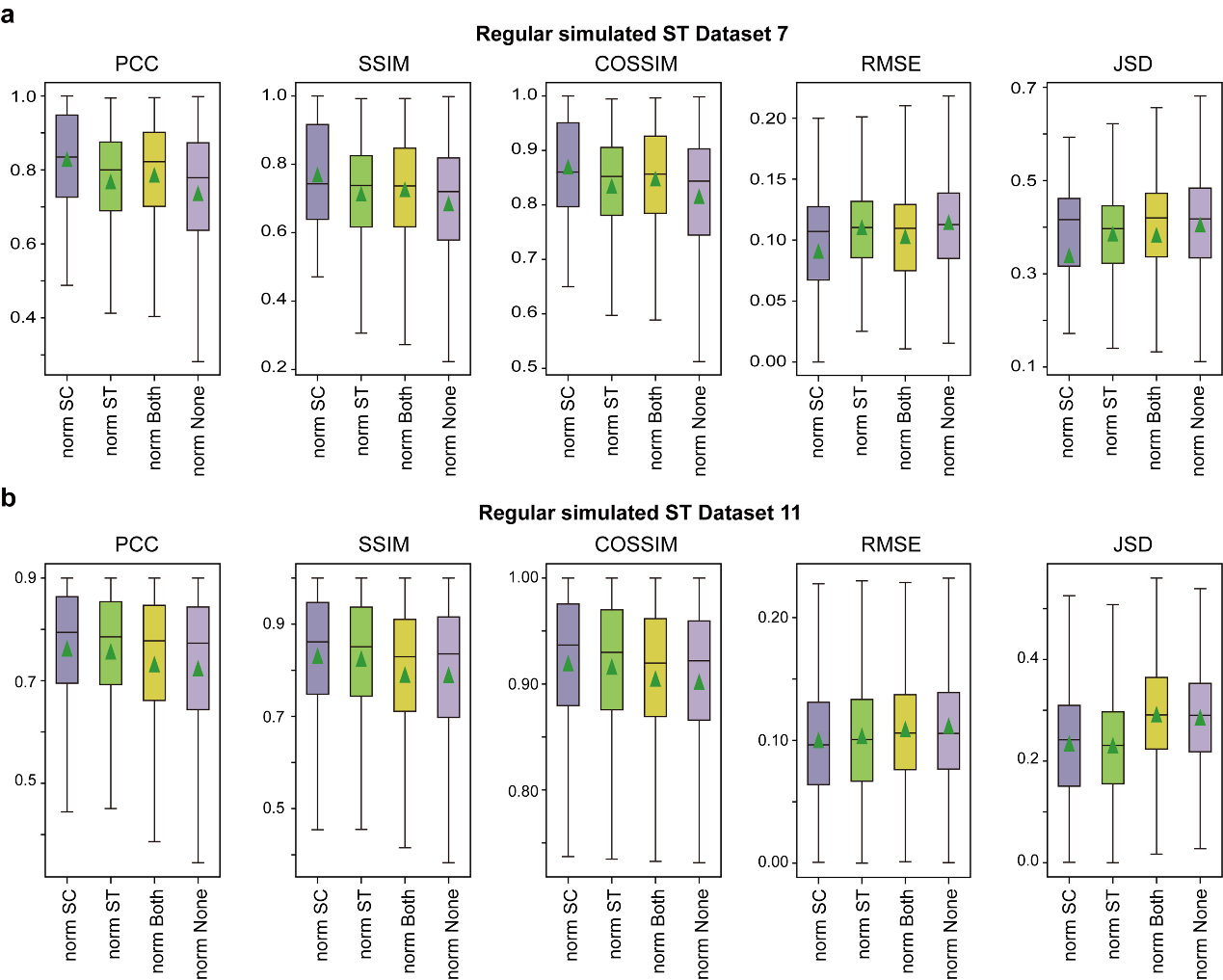


**Supplementary Figure 2. The performance of SpatialcoGCN using different data normalization strategies.** (**a**) Performance of SpatialcoGCN using different data normalization strategies on regular simulated dataset 7. (**b**) Performance of SpatialcoGCN using different data normalization strategies on regular simulated dataset 11.


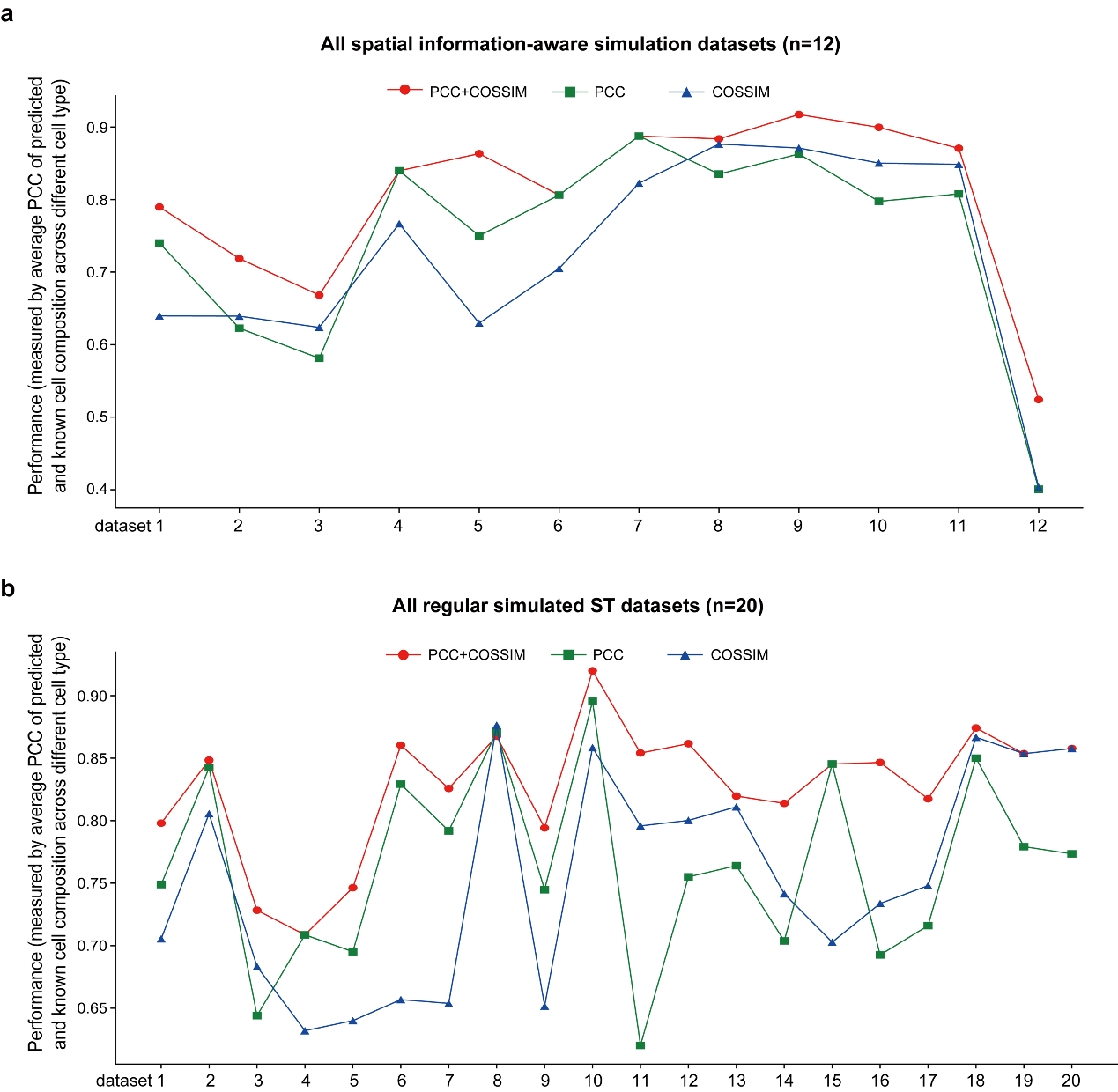


**Supplementary Figure 3. Ablation experiments about the similarity metrics used in loss function.** The ablation experiments compared the performances using PCC alone, using cosine similarity alone, and using both PCC and cosine similarity in the loss functions of the deep learning model. (**a**) Line charts comparing the performance across 12 spatial information-aware simulation datasets. (**b**) Line charts comparing the performance across 20 regular simulated ST datasets.


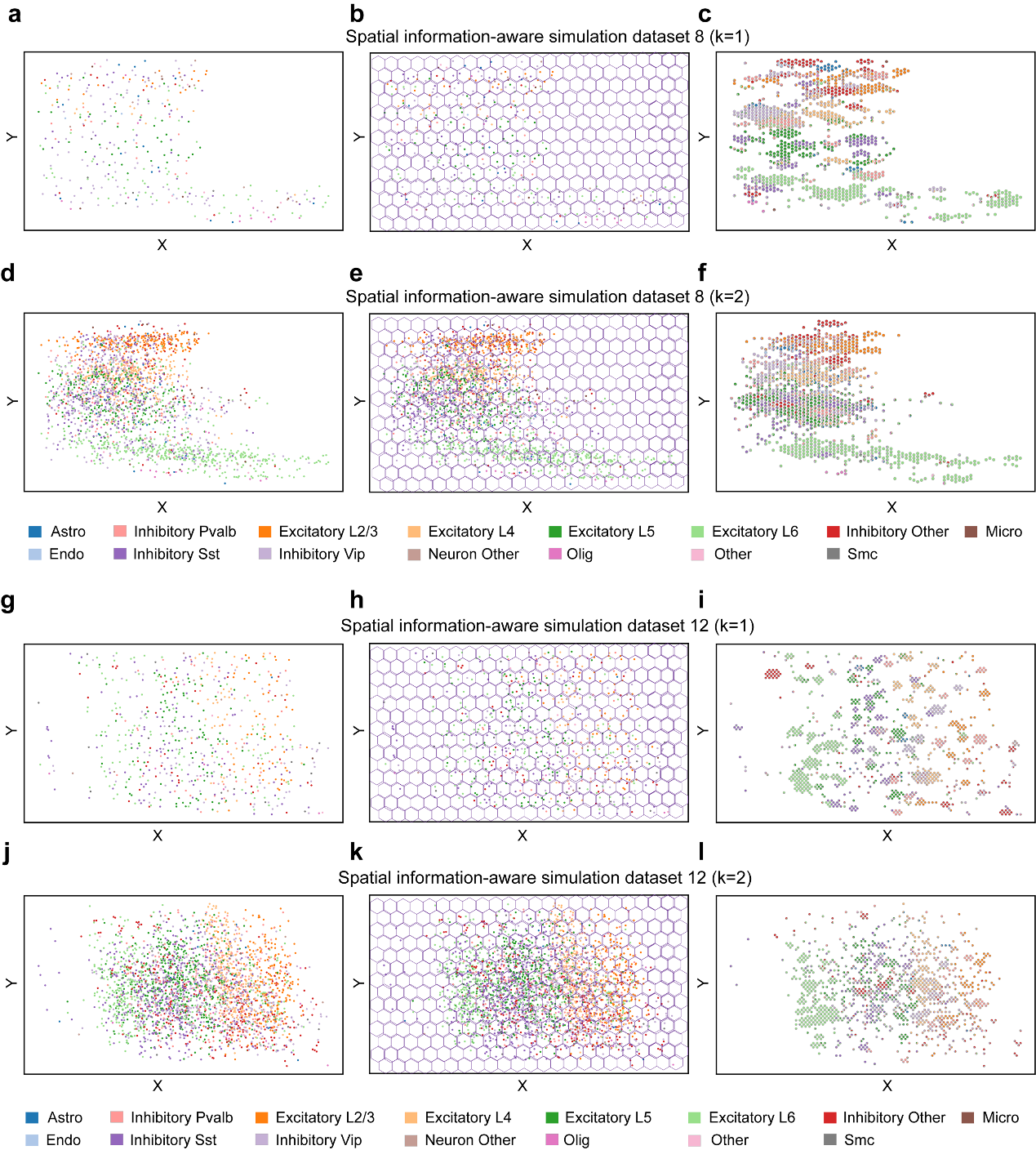


**Supplementary Figure 4. Sample illustration of the process and result in generating spatial information-aware dataset by SpatialcoGCN-Sim.** (**a**) Assigning each cell a 2D coordinate for dataset 8 using 1-nearest neighbor (k = 1) regression. (**b**) Matching the hexagonal grid on dataset 8 to represent low-spatial-resolution spots. The height of the hexagon is 80 pixels. (**c**) The generated spatial information-aware simulation dataset 8. (**d-f**) Same as (**a-c**), but using 2-nearest neighbor (k = 2) regression instead. A more obvious spatial organization can be observed when using 2-nearest neighbor. (**g**) Assigning each cell a 2D coordinate for dataset 12 using 1-nearest neighbor regression. (**h**) Matching the hexagonal grid on dataset 12 to represent low-spatial-resolution spots. The height of the hexagon is 200 pixels. (**i**) The generated spatial information-aware simulation dataset 12. (**j-l**) Same as (**g-i**), but using 2-nearest neighbor regression instead. A more obvious spatial organization can be observed when using 2-nearest neighbor.

**
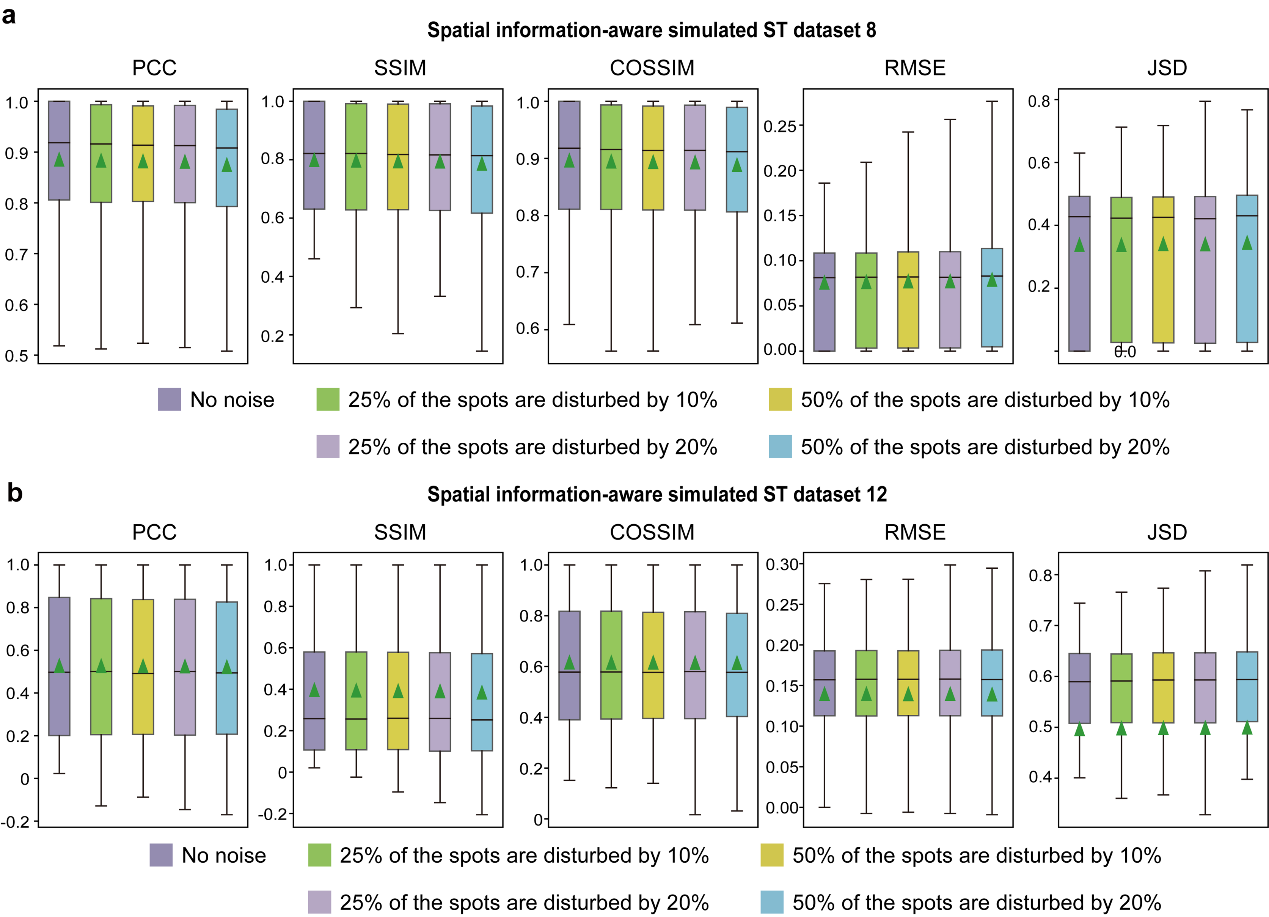
**

**Supplementary Figure 5. Evaluation of SpatialcoGCN on the noisy dataset.** (**a**) Performance of SpatialcoGCN on the spatial information-aware dataset 8 before and after the expression profile is disturbed by the neighbor spots to varying extents. (**b**) Performance of SpatialcoGCN on the spatial information-aware dataset 12 before and after the expression profile is disturbed by the neighbor spots to varying extents.

**
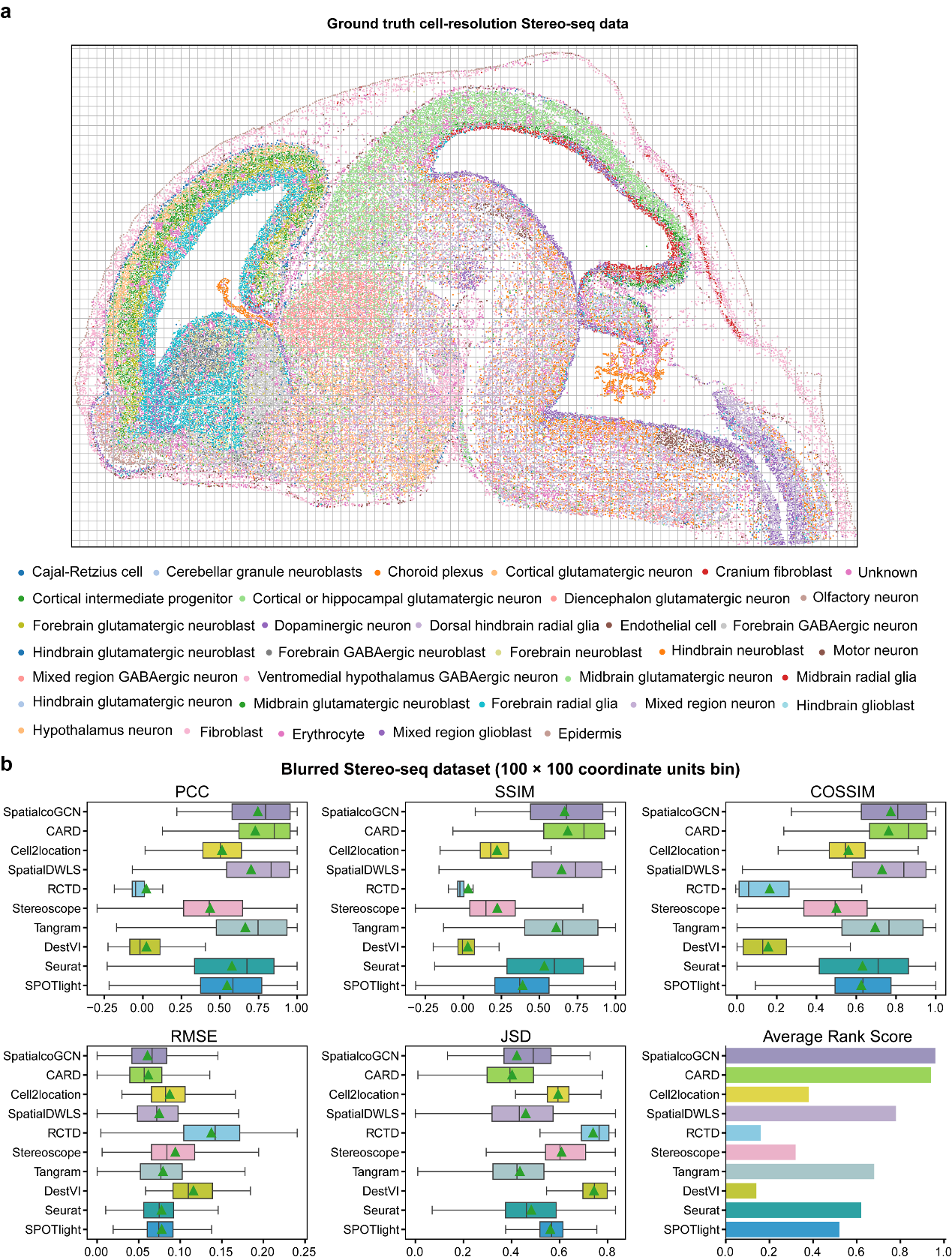
**

**Supplementary Figure 6. Evaluation of deconvolution performance on blurred Stereo-seq data.** (**a**) Ground truth cell-resolution Stereo-seq data of the mouse brain, which was further partitioned into 100 × 100 coordinate bins (bin100) to simulate the low-resolution data. Each dot is a single cell colored by its ground truth cell type label. The bins are indicated by the grey grids on the spatial map. (**b**) The boxplots of PCC, SSIM, COSSIM, RMSE, and JSD, along with the bar plot for ARS illustrating the prediction performance of cell type composition on the blurred Stereo-seq dataset.

**
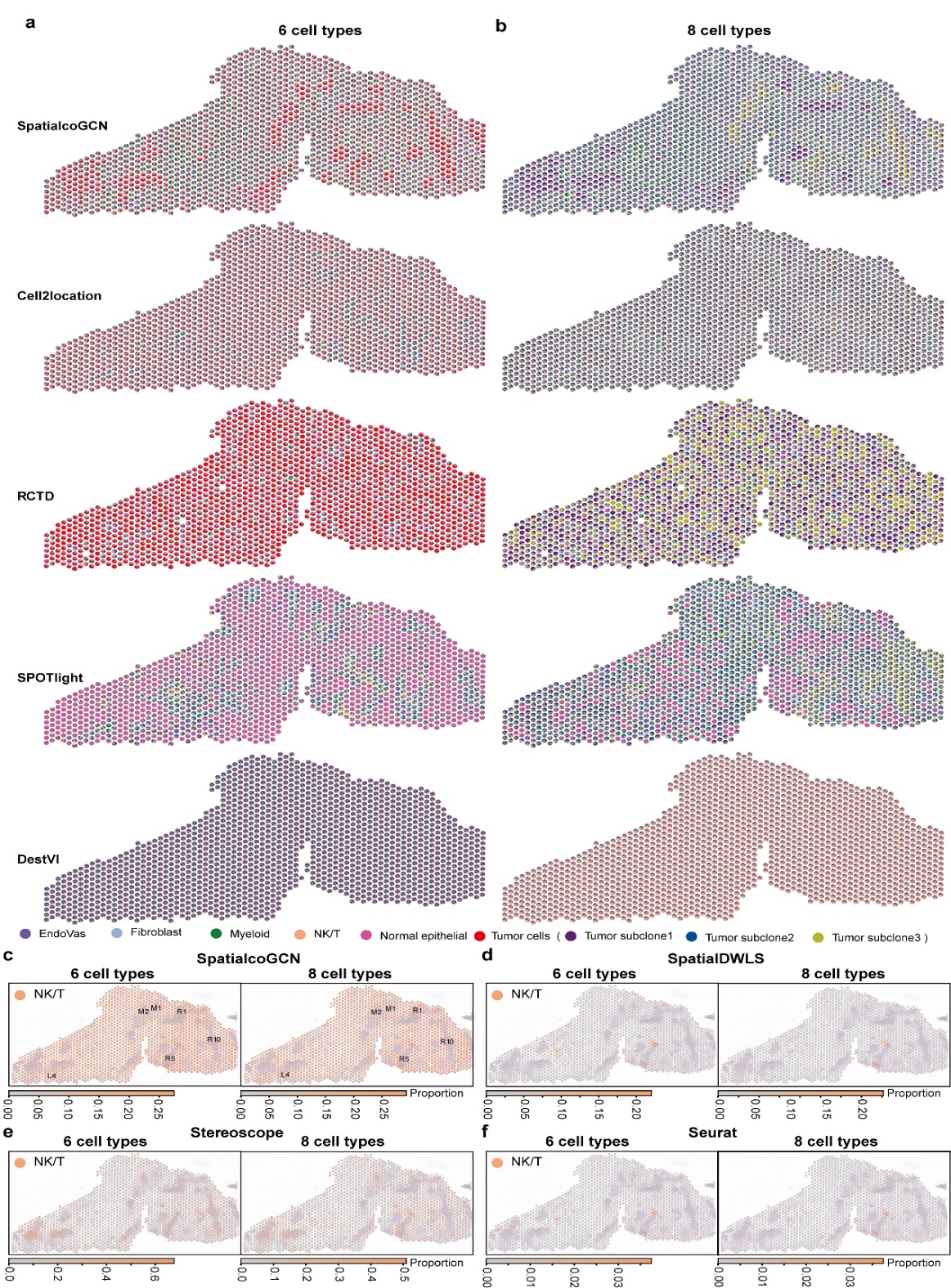
**

**Supplementary Figure 7. Comparison of deconvolution results by different methods on DCIS datasets, continued.** (**a-b**) The spatial distributions of cell type proportion predicted by spaitialcoGCN and other four deconvolution methods using (**a**) 6 cell types scRNA-seq reference and (**b**) 8 cell types scRNA-seq reference, respectively, are shown. Each pie represents the cell type proportions in each spot in the ST slide, and colors represent different cell types. (**c-f**) The spatial distribution of NK/T cells predicted by different methods using either the 6 cell type or the 8 cell type references. Spatial distribution of NK/T cells predicted by (**c**) SpatialcoGCN, (**d**) SpatialcoDWLS, (**e**) Stereoscope deconvolution, and (**f**) Seurat deconvolution, are shown respectively.

**
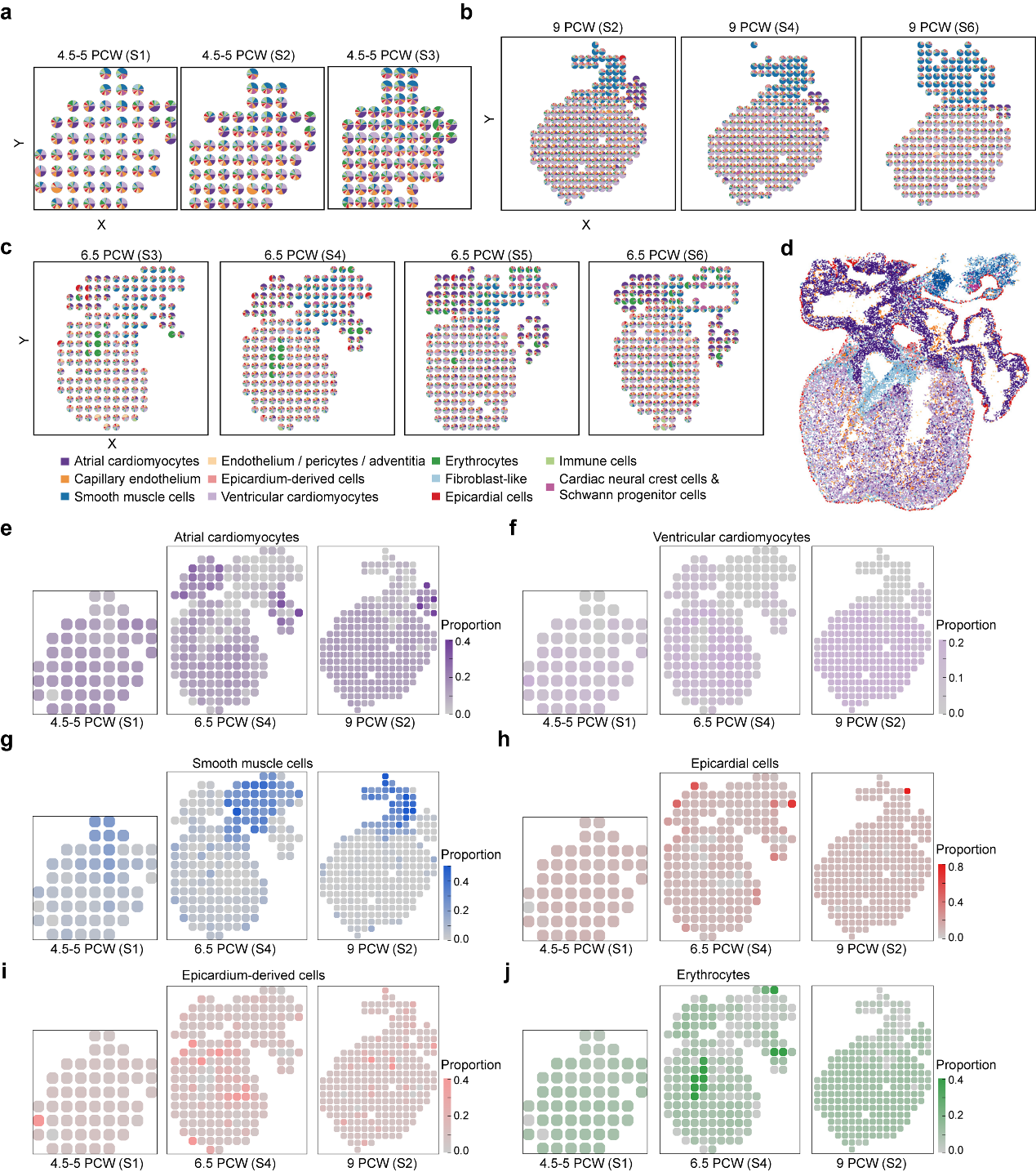
**

**Supplementary Figure 8. SpatialcoGCN’s deconvolution results on low-resolution developing human heart ST data.** (**a**) The deconvolution results of sample 1, 2 and 3 from 4.5–5 PCW. Each scatter represents a spot in the ST slide. The pie chart is used to reflect the proportions of different cell types in each spot. Colors represent different cell types. (**b**) The deconvolution results of sample 2, 4 and 6 from 9 PCW. (**c**) The deconvolution results of sample 3, 4, 5 and 6 from 6.5 PCW. (**d**) The reference spatial cell-type map created through the integration of ISS and scRNA-seq by the original study. (**e-j**), The resulting estimates of the spatial distribution of different cell types. One representative sample was selected from each developmental stage.

**
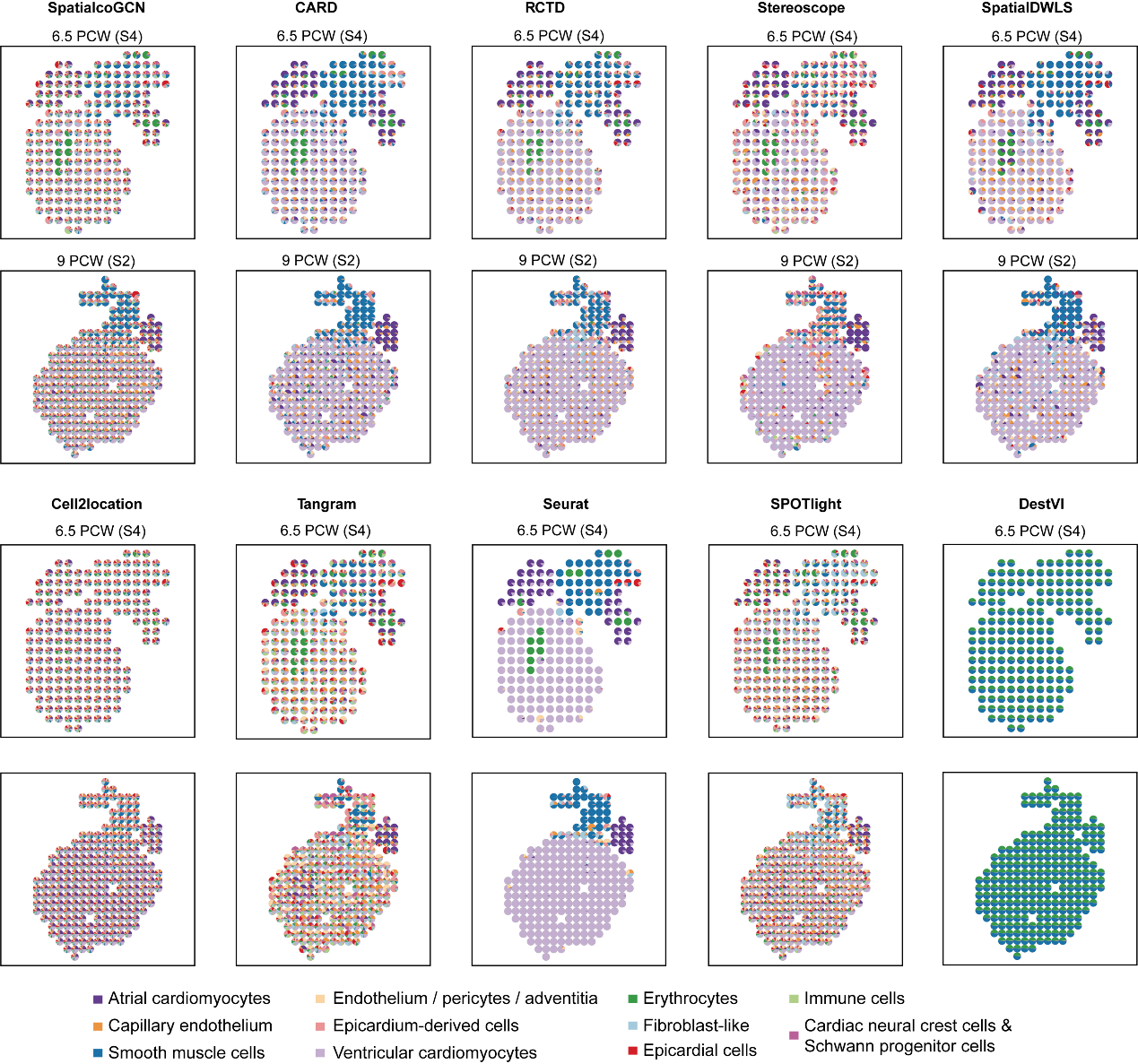
**

**Supplementary Figure 9. Comparison of deconvolution results on low-resolution developing human heart ST data by different methods.** The deconvolution results by SpatialcoGCN and other nine deconvolution method are shown. The sample 4 from 6.5 PCW and sample 2 from 9 PCW are used for the illustration.

**
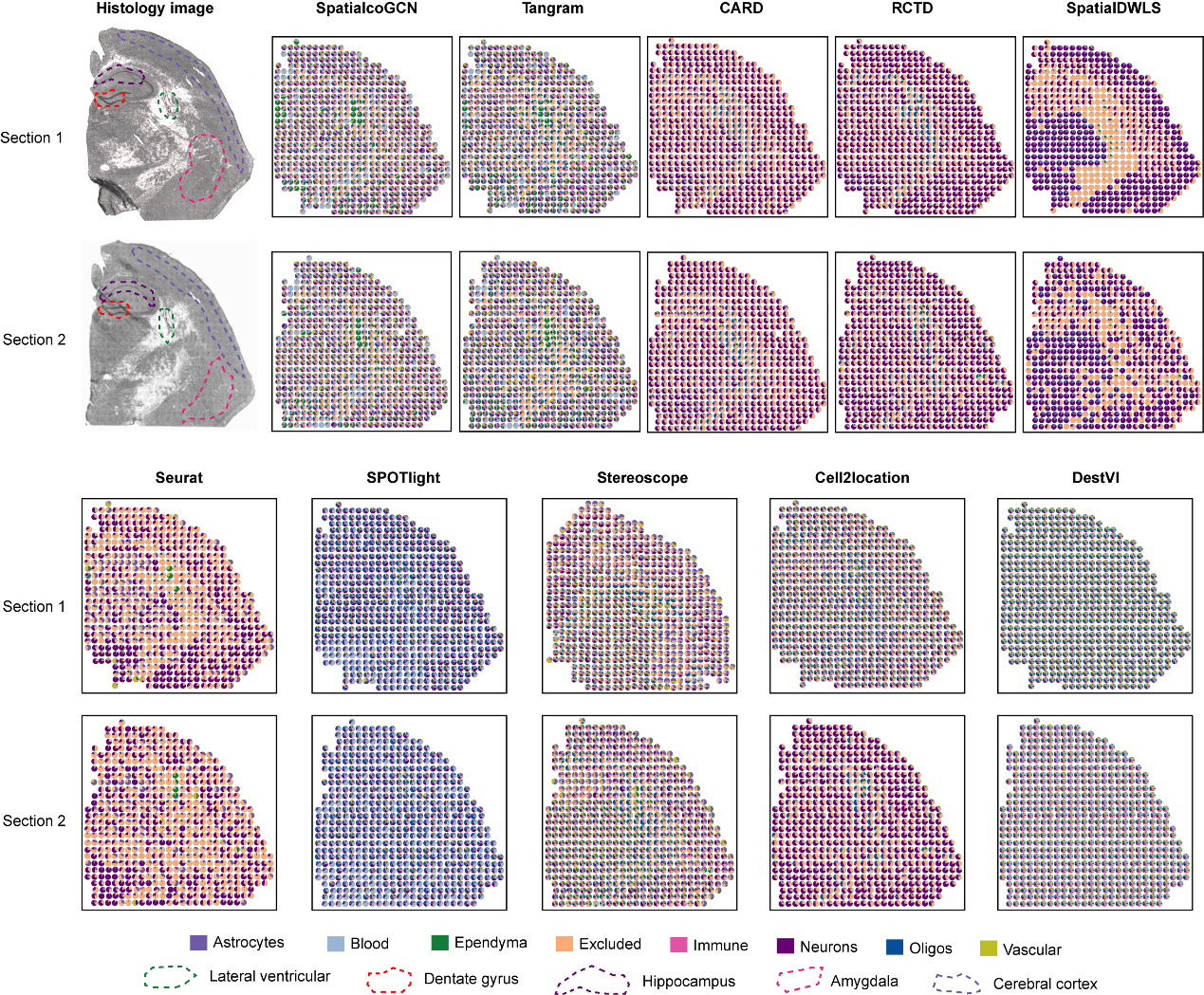
**

**Supplementary Figure 10. Comparison of deconvolution results on low-resolution mouse brain ST data by different methods.** The deconvolution results by SpatialcoGCN and other nine deconvolution method are shown. The matched histological images are also shown, on which the featured brain regions are annotated.

**
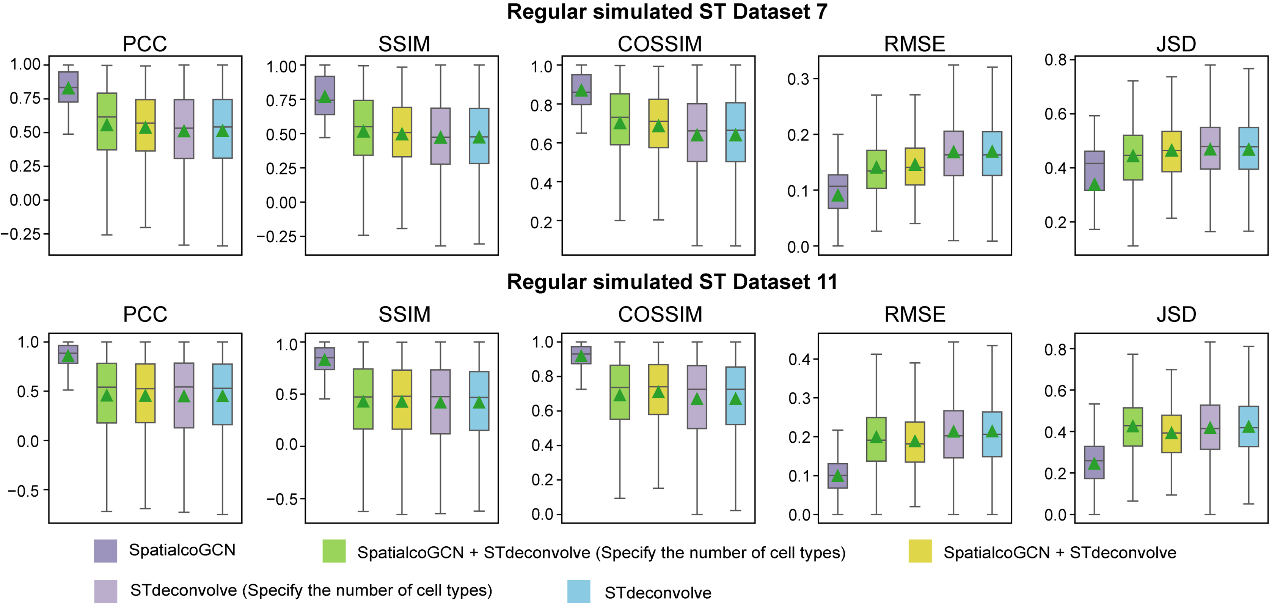
**

**Supplementary Figure 11. Performance of SpatialcoGCN incorporating reference-free deconvolution.** (**a**) The performance of SpatialcoGCN incorporating reference-free deconvolution on regular simulated ST dataset 7. (**b**) The performance of SpatialcoGCN incorporating reference-free deconvolution on regular simulated ST dataset 11. STdeconvolve first predicted the number of cell types when the number of cell types was unknown. The term “specify the number of cell types” means that we specified the known number of cell types when running STdeconvolve to avoid wrong results due to incorrect estimation of cell type number by STdeconvolve.

**
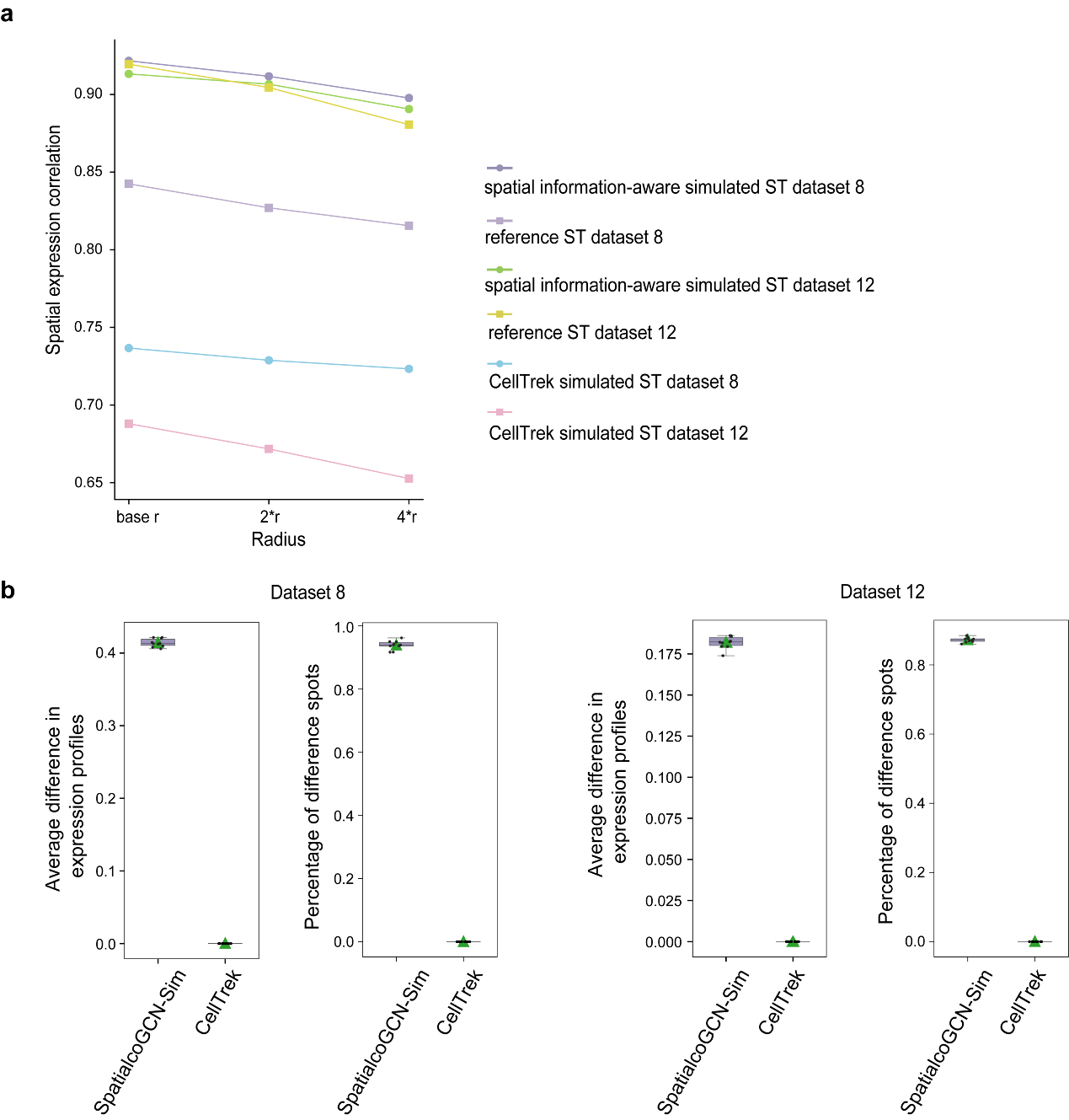
**

**Supplementary Figure 12. The comparison of spatial information-aware simulated ST dataset generated by SpatialcoGCN-Sim and CellTrek.** (**a**) The spatial expression correlation of spatial information-aware simulated ST datasets by SpatialcoGCN-Sim, reference ST datasets, and CellTrek simulated ST datasets. (**b**) Comparison of the diversity of simulated ST data (i.e., whether the expression pattern in different simulated data show prominent divergences).
